# Simulations of rate of genetic gain in dry bean breeding programs

**DOI:** 10.1007/s00122-023-04244-x

**Published:** 2023-01-20

**Authors:** Jennifer Lin, Vivi Arief, Zulfi Jahufer, Juan Osorno, Phil McClean, Diego Jarquin, Valerio Hoyos-Villegas

**Affiliations:** 1 Department of Plant Science, McGill University, Montreal, Canada; 2 University of Queensland, Brisbane, Australia; 3 North Dakota State University, Fargo, ND USA; 4 University of Florida, Gainesville, FL USA

## Abstract

**Key message:**

A reference study for breeders aiming at maximizing genetic gain in common bean. Depending on trait heritability and genetic architecture, conventional approaches may provide an advantage over other frameworks.

**Abstract:**

Dry beans (*Phaseolus vulgaris* L.) are a nutrient dense legume that is consumed by developed and developing nations around the world. The progress to improve this crop has been quite steady. However, with the continued rise in global populations, there are demands to expedite genetic gains. Plant breeders have been at the forefront at increasing yields in the common bean. As breeding programs are both time-consuming and resource intensive, resource allocation must be carefully considered. To assist plant breeders, computer simulations can provide useful information that may then be applied to the real world. This study evaluated multiple breeding scenarios in the common bean and involved five selection strategies, three breeding frameworks, and four different parental population sizes. In addition, the breeding scenarios were implemented in three different traits: days to flowering, white mold tolerance, and seed yield. Results from the study reflect the complexity of breeding programs, with the optimal breeding scenario varying based on trait being selected. Relative genetic gains per cycle of up to 8.69% for seed yield could be obtained under the use of the optimal breeding scenario. Principal component analyses revealed similarity between strategies, where single seed descent and the modified pedigree method would often aggregate. As well, clusters in the direction of the Hamming distance eigenvector are a good indicator of poor performance in a strategy.

**Supplementary Information:**

The online version contains supplementary material available at 10.1007/s00122-023-04244-x.

## Introduction

With increasing global populations and the current implications of climate change, meeting demands for food security while instilling sustainable practices is imperative. In addition to providing high-quality nutrients for both human and animal consumption, legumes are remarkably sustainable to grow. They can reduce greenhouse gas emissions and can improve soil fertility by increasing carbon and nitrogen content and availability (Stagnari et al. 2017). Dry beans are an important legume crop grown in many developing countries that greatly contribute to the energy and nutritional intake in low-income regions (Siddiq and Uebersax 2012; Stagnari et al. 2017). Rich in proteins, carbohydrates, fibers, vitamins, and minerals, dry beans offer health benefits that are unrivaled. Research has shown that dry beans contain soluble fibers that can lower serum cholesterol, which in turn improves coronary health. Dry beans are also excellent for metabolic control. They lead to miniscule increases in blood glucose and insulin, making them highly suitable for diabetic individuals. Due to the nutritional quality of dry beans, they may be also used to combat obesity (Geil and Anderson 1994).

Increasing dry bean yield is of importance for both developed and developing countries that rely on this legume. The main constraints to increasing yield are biotic and abiotic stresses. Breeding for tolerance to drought stress, heat stress, cold stress, and low nutrient stress is important in particularly in areas with harsher growing conditions. Breeding for biotic stresses is also crucial, as dry beans are susceptible to several diseases that can severely limit yield. In temperate growing regions, the most common diseases include common bacterial blight, halo blight, rust, and white mold. Some breeders are also interested in agronomic traits that may improve yield. For example, selecting for upright plant architecture can facilitate harvest and reduce vulnerability to disease, which can indirectly benefit yield (Soltani et al. 2016). When it comes to dry bean breeding, the market class must be taken into consideration. Market classes broadly categorize dry beans based on their physical and physiological properties, including color, size, and shape on the basis of consumer preferences. For example, the pinto bean market class consists of small to medium sized seeds with a mottled brown color on a cream background. For certain market classes, enhancing yield may be difficult due to the yield component compensation, where some yield components are negatively correlated with each other (Adams 1967). In general, plant breeders will develop strategies that are applicable to their growing region and market class of choice. Traditionally, dry bean breeders have used early generation testing and visual selection to improve yield. However, these strategies have their limitations, namely in that yield testing is extremely costly and laborious. Thus, it may be worthwhile to delay yield testing until later generations (Kelly et al. 1998). Other traits of interest for improvement include those that are consumer driven. In developing countries, faster cooking time is desired since fuel is often in short supply. To fight malnutrition in low-income areas, breeding programs may focus on improving nutrient content, such as zinc and iron. In developed countries, canning quality is an important trait for improvement (Beaver and Osorno 2009).

An important goal in plant breeding is achieving genetic gain (∆*G*), which is the rate of change in the mean of a trait being selected for in a population (Falconer 1960; Moose and Mumm 2008; Sun et al. 2011). The equation for genetic gain is as follows:

1 $$\Delta G = \frac{{h^{2} i\sigma_{a} }}{L}$$

where, *h*^2^ refers to the narrow-sense heritability, σ_a_ is the additive variance, *i* is the selection intensity, and *L* is the generation interval (Sun et al. 2011). Equation 1 highlights the different factors that contribute to genetic gain. Typical dry bean breeding programs take up to 10 years and require extensive resources in the process. Due to the long-term commitment, the decisions that go into a breeding program must be carefully considered. For this purpose, plant breeders can make use of computer simulations to assist in decision making.

Due to the complexity of breeding programs, the breeder’s equation is used as a basis for which the simulation studies are conducted. Each component of the equation may be evaluated to determine their impact on genetic gain. Simulation studies make it possible to acquire information that could not be obtained empirically. Furthermore, multiple different breeding scenarios can be evaluated in a short period of time, allowing for various comparisons to be made. The data obtained from studies may be used to help breeders decide where emphasis should be placed when designing a breeding program. The goal of any breeding program is to maximize genetic gain in the shortest amount of time. The heritability of a trait will impact a breeding program. Traits with a higher heritability can result in greater genetic gain. The selection intensity will also impact the genetic gain.

The overall aim of this study was to explore the feasibility of driving genetic gain forward in the presence of multiple reported QTLs segregating in the population. The specific objectives of this study were to: (1) simulate a baseline of selection strategies and determine differences in genetic gain performance; (2) test whether changes in initial parental population size and trait heritability lead to increased genetic gain and percentage of fixed favorable alleles.; (3) test if genomic selection and speed breeding outperform conventional breeding frameworks in terms of long-term genetic gain and allele fixation rate.

## Materials and methods

We used the QuLinePlus (Hoyos-Villegas et al. 2019) module in the QU-GENE platform to simulate the outcomes of different breeding frameworks, selection strategies and initial parental population sizes on three traits of varying heritability. The focus of this paper will be on stochastic modeling of genetic gain across two agronomic traits (yield and days to flowering) and a biotic stress (white mold tolerance). The three traits were chosen on known differing heritability levels examined with seed yield as low h^2^, days to flowering as moderate h^2^, and white mold tolerance as high *h*^2^. Three breeding frameworks, five selection strategies and three initial parental population sizes were considered. We provide a short description of each breeding scenario (selection strategies and frameworks) and their implementation in QuLinePlus.

### Breeding frameworks

In recent years, new proposed breeding frameworks have emerged, namely, speed breeding (Watson et al. 2018) and genomic selection (Meuwissen et al. 2001) that have the potential to outperform conventional/phenotypic breeding frameworks. Speed breeding can circumvent the developmental constraints in plants, thus reducing the total length of a breeding program and subsequently allowing for greater genetic gains per year. Genomic selection uses models that predict phenotypes based on all markers across a genome to select on genotypes. This allows for selection to take place before at the seedling stage. Using genomic selection, a plant breeder may genotype and cull poor performing lines, saving time and resources otherwise required to phenotype each line.

#### Speed breeding

Speed breeding is a technique used to increase the rate of development in crops and as a result, decrease generation times (Watson et al. 2018). Methods in speed breeding typically involve lengthening the photoperiod, with 22 h of light and 2 h of dark. Speed breeding has been successfully implemented in a number of crop species, including wheat, barley, chickpea, canola, and pea (Watson et al. 2018). In dry beans, speed breeding may be used to advance plants from the crossing block to the F_4_ generation in a single year, significantly cutting down the duration of a breeding program. In the simulation, a cycle is defined as the period starting from the crossing stage until the F8 generation where a variety is selected. If it is assumed that each generation in conventional breeding requires one year, then the cycle length from crossing to F8 would be 8 years. As crossing can be done in a greenhouse over winter, it can be excluded from the cycle length. Under speed breeding, crossing to F4 is achieved in a single year instead of 4 years, which results in a cycle length of 5 years. Relative to conventional breeding, speed breeding is 1.6 times faster. To account for this difference, the cycle length was adjusted post-run using R version 4.0.2 (R Core Team 2020).

#### Genomic selection

First described by Meuwissen et al. (2001), genomic selection involves estimating the effects of all molecular markers (e.g., single nucleotide polymorphisms, SNPs) and selecting on individuals based on their genomic estimated breeding value (GEBV) (Michel et al. 2016). To implement genomic selection, a parental population was simulated and used as the training population. As QU-GENE simulates populations that are in Hardy–Weinburg equilibrium, a different program was used instead. SimuPOP is a program that is capable of simulating a population with adequate linkage disequilibrium (LD), which is necessary for genomic selection to be successfully utilized. Using this training population, QuLinePlus was run without any selection to simulate marker and phenotypic data. The ‘rrBLUP’ package version 4.6.1 was used to determine the marker effects of the SNPs. From there, QuLinePlus was run again, and selection was conducted based on the marker effects. The selection candidates were the progeny of the parental/training population.

### Selection strategies

Several selection strategies for self-pollinated crops were tested. Conventional selection strategies included bulk breeding, single seed descent, mass selection, the pedigree method, and the modified pedigree method. These conventional strategies relied solely on phenotypic selection.

#### Mass selection

**Fig. 1:**
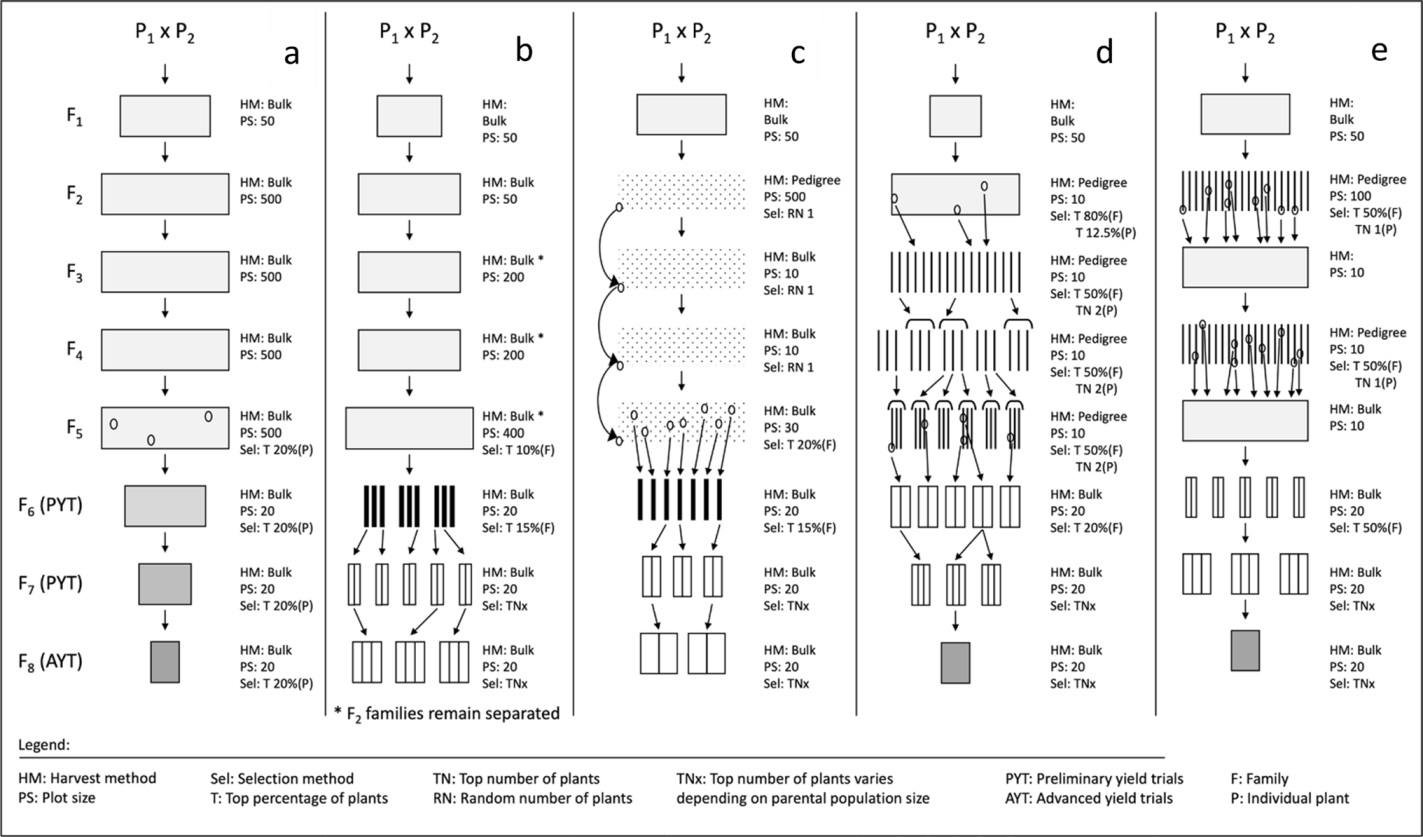
Selection strategies simulated in QuLinePlus. (**a**) Mass selection, (**b**) Bulk breeding, (**c**) Single seed descent, (**d**) Pedigree and (**e**) Modified pedigree

Mass selection is the oldest form of crop improvement and was carried out by farmers long before the concepts of Mendelian genetics and the development of pure-lines were commonplace (Fehr 1987). In mass selection, desirable plants are selected from an entire population and a sample of the seeds collected form the next generation of plants. This process is repeated for several generations until the multi-environment trial phase (Fig. 1a). The key purpose of mass selection is to improve the average of the baseline population (Acquaah 2009). However, this improvement is typically constrained by the genetic variability of the initial population. Mass selection may be used to develop varieties from a hybridized population. In this approach, undesirable plants are removed from the population. In some cases, mass selection is performed to purify lines. When deciding to use mass selection, the trait heritability should be considered, as high heritability traits are much more successful (Fehr 1987).

#### Bulk breeding

Bulk breeding is a strategy that relies on natural selection in early generations to remove low performing genotypes (Fehr 1987). Artificial selection is only conducted in later generations once a high amount of homozygosity is present in the F_2_ derived lines. The process begins with the crossing of two parents and continues with the bulking of each segregating generation. Once sufficient homozygosity has been achieved, the plants will be assessed and those with the desired trait cassette will be selected. Following this, multi-environment testing will take place, and superior lines will be identified (Fig. 1b). One of the major criticisms of bulk breeding is that it promotes competition between genotypes, so there is a possibility that a desirable genotype is outcompeted by an undesirable genotype. Another concern is that some traits that persist due to natural selection have no agricultural benefit. Nevertheless, bulk breeding is still less labor intensive and cheaper than some other strategies and it allows plant breeders to make and assess more crosses (Acquaah 2009).

#### Single seed descent

Single seed descent is a method that attempts to achieve homozygosity in the shortest amount of time (Acquaah 2009). The objective is to advance as many F_2_ plants as possible to the F_5_ generation. This is done by taking one random seed from each plant to advance to the next generation until yield trials (Fig. 1c). Not only does this method require fewer resources, but it is also possible to advance multiple generations in a single year by using greenhouses and winter nurseries. Selection only takes place in later generations once adequate homozygosity is reached. Unlike bulk breeding, earlier generations do not undergo natural selection and each F_2_ plant is equally represented, meaning each generation has more genetic diversity. The main disadvantage is that not every seed will germinate or be sampled from the population. Thus, not all F_2_ individuals will be represented in the later generations.

#### Pedigree method

The pedigree method is a strategy whereby pedigree relationships are carefully recorded; thus, any individual plant can be easily traced back to an F_2_ plant. The pedigree method differs from the previous methods in that artificial selection takes place in segregating populations. Selection occurs at each generation and begins at the F_2_ generation. Individual F_2_ plants that were selected are grown in rows, forming the F_3_ generation. Each row can also be referred as a family. Individual plants within rows or even entire rows may be selected (Fig. 1d). This continues until there is an acceptable level of homozygosity (Fehr 1987). A benefit to using the pedigree method is that thorough pedigree, and other valuable genetic information is now available to plant breeders through plant breeding software, which facilitates the collection and analysis of pedigree and genetic data, in addition to the processing of bioinformatic data (Agronomix software). Furthermore, the records may be used to better select lines that carry a desirable trait. The main concern with the pedigree method is that it is resource demanding. Record-keeping is time-consuming and progeny rows can take up lots of space (Acquaah 2009).

#### Modified pedigree method

The modified pedigree is a method that takes into consideration the importance of inbreeding before making selections. This is because genetic variance will increase between lines, but decrease within lines (Brim 1966). Individual plant and row selections take place in the F_2_ and F_4_ generations, where plants are grown in their target growing region. This strategy also makes use of winter nurseries in the F_3_ and F_5_ generation, where selected lines are harvested in bulk (Fig. 1e). In short, the use of winter nurseries in the modified pedigree method saves time and resources, as harvesting plants in bulk is easier to manage. Meanwhile, it simultaneously allows plants to achieve homozygosity in less time (Acquaah 2009). This method has most recently been used for breeding a rust resistant variety of black bean (Osorno et al. 2021).

### QU-GENE simulation workflow and simulation files

**Fig. 2:**
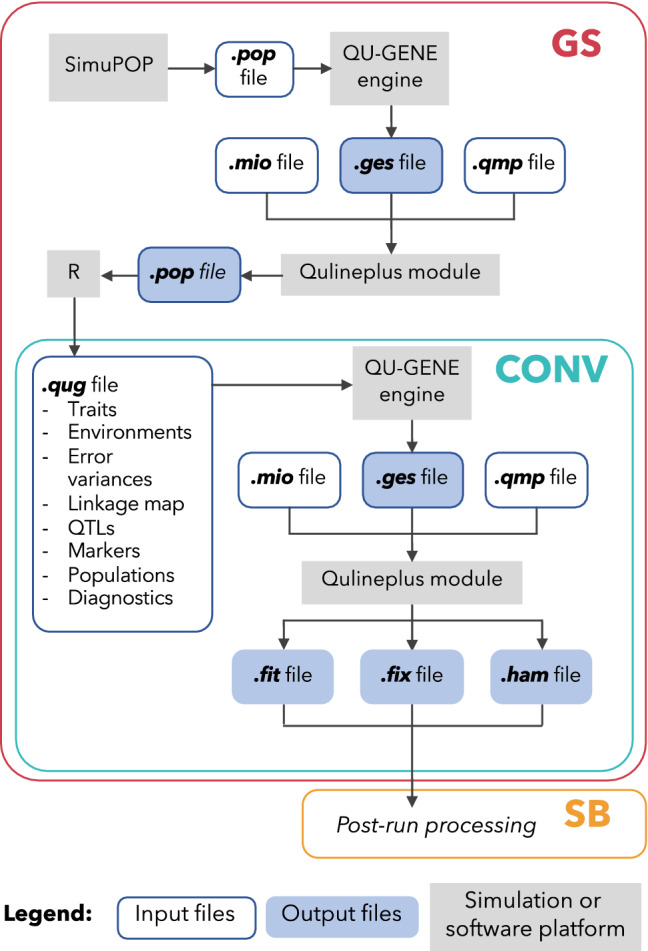
QU-GENE simulation workflow for simulation of breeding frameworks. Genomic selection (GS), conventional (CONV), and speed breeding (SB)

Simulation was conducted to compare four different combinations of initial parents, three different traits, three breeding frameworks, and five selection strategies. Each simulation consisted of 10 cycles with 50 runs/cycle. A summary of the simulation criteria is displayed in Table 1. All the files required by the simulation can be found on the GitHub page https://github.com/McGillHaricots/peas-andlove/tree/master/Simulation-files or via requests to the corresponding author. Figure 2 shows the workflow in QU-GENE for the simulation of conventional breeding, as well as the new proposed breeding frameworks, which require additional steps.

**Table 1:** Simulation criteria

Cycles	Runs	Parents	Traits	Environments	Framework	Strategies
10	50	15, 30, 60, 100	DF, WM, SY	Nursery, Winter Nursery, Field	Conventional, Speed breeding, Genomic selection	Mass selection, Bulk breeding, Single seed descent, Pedigree method, Modified pedigree method

Briefly, the file required by the QU-GENE engine is the. *qug* file, which contained the following: traits, environments, error variances, linkage map, QTLs, markers, populations, and diagnostics. In terms of traits, the three simulated traits were days to flowering, white mold tolerance, and seed yield. The simulation also involved three environments: nursery, winter nursery, and field. These three environments were included for the purposes of making the simulation more realistic. Based upon the literature, trait heritabilities varied depending on the environment. In the real world, plant breeders would make use of nurseries and winter nurseries, which may have different heritabilities compared to the field. As a result, these environments were included to create a more realistic simulation but were not directly investigated. To appropriately simulate the traits, the user must provide information on the environmental effects for the traits. This may be in the form of either within- or among-plot variances. Heritability estimates were provided as within-plot variances and are summarized in Table 2. The linkage map, QTLs, and markers described in an upcoming section were included in the .*qug* file. The diagnostic indicated that the file was error free and was able to be run in the QU-GENE engine.

**Table 2:** Narrow-sense heritability (h2) estimates for three traits in three environments

Trait	Environment	h^2^ estimate	Reference
DF	Nursery	0.67	Singh et al. (1990)
	Winter nursery	0.68	Nienhuis and Singh (1988)
	Field	0.92	Atuahene-Amankwa et al. (2004)
WM	Nursery	0.33	Carneiro et al. (2011)
	Winter nursery	0.65	Carvalho et al. (2013)
	Field	0.78	Miklas et al. (2001)
SY	Nursery	0.21	White and Singh (1991)
	Winter nursery	0.29	Mendes et al. (2008)
	Field	0.70	Kolkman and Kelly (2002)

*DF* days to flowering (in days), *WM* white mold tolerance (in disease incidence), *SY* seed yield (in kg/hectare)

Since QU-GENE simulates error variances based on the per plant broad-sense heritability, it was necessary to calculate these values based on the per plot heritability estimates reported in the literature (Atuahene-Amankwa et al. 2004; Carneiro et al. 2011; Carvalho et al. 2013; Kolkman and Kelly 2002; Mendes et al. 2008; Miklas et al. 2001; Nienhuis and Singh 1988; Singh et al. 1990; White and Singh 1991). While QU-GENE uses broad-sense heritability, narrow-sense heritability estimates were provided to QU-GENE, as only additive effects were considered among the QTL. This is because narrow-sense heritability approximates broad-sense heritability when the dominance effects are zero. In the simulation setup, all QTLs were defined as having additive effects, thus supplying narrow-sense heritability estimates in place of the broad-sense heritability estimates would not have an impact on the results. The following equation was used to determine the per plant broad-sense heritability:

2 $$H_{{{\text{per}}\; {\text{plant}}}}^{2} = \frac{{V_{g} }}{{V_{g} + \left( {\frac{{V_{e} }}{y} \times n} \right)}}$$

where *Vg* is the genetic variance, the phenotypic variance $V_{p} = V_{g} + \left( {\frac{{V_{e} }}{y} \times n} \right) = \frac{1}{{h^{2}_{{{\text{per}} \;{\text{plot}}}} }}$ **,** the error variance $V_{e} = V_{p} - V_{g}$, *n* is the plot size (number of plants), and *y* is the year.

The.*qmp* file included information on the selection strategies to be simulated. For each strategy, one cycle consisted of 8 generations, with selection occurring at different stages. As a closed system was being simulated, initial and final family sizes were the same. It included general information such as the number of strategies, the number of runs, and the number of cycles that were completed. It also included information specific to each selection strategy such as propagation type, generation advance method, number of replications, plot size, number of testing locations, and selection strategy (e.g. within family, among family, etc.). The propagation type indicated how the selected individuals from the previous generation were to be propagated to generate the individuals in the current generation. This experiment only considered “self” (self-pollination) and “clone” (asexual) as the propagation type. Although *Phaseolus vulgaris* L. does not have an asexual reproduction route, the term “clone” was used in QuLinePlus to migrate individuals from one breeding cycle to the next without changing their genetic composition. The term "clone” was also used to extract individuals sampled from the parent population into the first generation of the first breeding cycle. The generation advance method indicated how the selected plants were harvested. This experiment used the following generation advances: “pedigree”, “bulk”, and “superbulk”. “pedigree” meant plants were harvested individually, and each plant would result in a family in the next generation. “bulk” involved harvesting all plants in a family together, with no mixing of families. Finally, in “superbulk”, all plants were harvested to form one population regardless of family. Details for each strategy are shown in Supplementary Table 1.

The QuLinePlus module was used to simulate the selection strategies. It is capable of simulating both self-pollinating and cross-pollinating species (Hoyos-Villegas et al. 2019). With regards to GS, selection was carried out based on the marker effects, and was done in the F_5_ generation for mass selection, bulk breeding, and single seed descent, and implemented in the F_2_ and F3:4 generations for the pedigree method and the modified pedigree method, respectively. Output files obtained from the QU-GENE engine were used as input files for QuLinePlus. All simulations were performed remotely on servers provided by Compute Canada (Digital Research Alliance of Canada 2020). Access to remote servers required establishing a secure shell via the terminal on MacOS. To browse and manipulate files, the cloud storage browser Cyberduck was used (iterate GmbH 2020).

### Linkage map and QTLs

The common bean consensus linkage map reported by Galeano et al. (2011) was used for this study. It was developed from the recombinant inbred lines from three different Mesoamerican intra-gene pool linkage mapping populations. The consensus linkage map was made up of 1010 markers and had a map length of 2041 cM over 11 linkage groups. Each linkage group had an average of 91 markers. Since more markers could be identified through the combining from multiple segregating populations than can be obtained from a single population, and greater coverage can be achieved, this consensus map was selected for conducting the simulations. In contrast to a physical map, a genetic consensus map was used to provide QU-GENE with recombination fraction information for simulation.

A total of 38 QTLs found in the literature were considered for this study. Specifically, 11 seed yield QTLs, 8 white mold disease incidence QTLs, and 19 days to flowering QTLs were selected (Table 3). Seed yield QTL effect sizes ranged from −36.91 to −197.46 kg/ha. QTL effect sizes for white mold disease QTLs ranged from 3.16 to −7.2. QTL effect sizes in days to flowering ranged from 0.68 to −1.21 days. The reported QTL effect sizes were the additive genetic effects that could be attributed to having one of the alleles. In the simulation, it was assumed that having the alterative allele would lead to an equal but opposite effect. That is, if at locus A, the possible genotypes were AA, Aa, and aa, and allele A had an effect size of *s*, then it was assumed that AA would have effect size 2* s*, Aa would have effect size 0, and aa would have effect size −2 s*.*

**Table 3:** Description of QTLs used in the simulation

Trait	QTL name	Linkage group	Position (cM)	Effect size	Mapping population	Reference
Days to Flowering (Days)	DF41	4	167.11	0.68	DOR 364 × BAT 477	Diaz et al. (2017)
	DF51	5	45.21	0.45	DOR 364 × BAT 477	Diaz et al. (2017)
	DF52	5	56.71	0.49	DOR 364 × BAT 477	Diaz et al. (2017)
	DF53	5	82.21	0.46	DOR 364 × BAT 477	Diaz et al. (2017)
	DF54	5	105.21	0.43	DOR 364 × BAT 477	Diaz et al. (2017)
	DF11a	11	96.51	−0.6	DOR 364 × BAT 477	Diaz et al. (2017)
	DF11b	11	108.71	−0.49	DOR 364 × BAT 477	Diaz et al. (2017)
	EM86	2	21.6	0.57	Bunsi × Newport	Ender and Kelly (2005)
	EM78	7	1.1	−0.6	Bunsi × Newport	Ender and Kelly (2005)
	EM550	7	13.6	−0.96	Bunsi × Newport	Ender and Kelly (2005)
	EM223	7	8.6	−1.21	Bunsi × Newport	Ender and Kelly (2005)
	DF121	1	51	0.02	SER48 × Merlot	Hoyos-Villegas et al. (2016)
	DF122	1	62	−0.69	SER48 × Merlot	Hoyos-Villegas et al. (2016)
	DF111	1	47	−0.62	SER48 × Merlot	Hoyos-Villegas et al. (2016)
	DF13	1	19	0.12	SER48 × Merlot	Hoyos-Villegas et al. (2016)
	DF112	1	40	0.03	SER48 × Merlot	Hoyos-Villegas et al. (2016)
	DF123	1	59	−0.66	SER48 × Merlot	Hoyos-Villegas et al. (2016)
	DFmn1	1	16.9	−0.8	AN-37 × P02630	Hoyos-Villegas et al. (2015)
	DFmn2	1	105.7	−0.8	AN-37 × P02630	Hoyos-Villegas et al. (2015)
White Mold Severity (1–9 Score)	WM2010	3	91.5	−7.2	AN-37 × P02630	Hoyos-Villegas et al. (2015)
	WM31	3	111.1	−4	AN-37 × P02630	Hoyos-Villegas et al. (2015)
	DSI1	2	8	3.15	Bunsi × Newport	Ender and Kelly (2005)
	DSI2	2	21	−2.66	Bunsi × Newport	Ender and Kelly (2005)
	DSI3	5	27.7	3.16	Bunsi × Newport	Ender and Kelly (2005)
	DSI4	7	8.6	−4.17	Bunsi × Newport	Ender and Kelly (2005)
	DSI5	7	14.8	−4.01	Bunsi × Newport	Ender and Kelly (2005)
	DSI6	8	1.4	2.93	Bunsi × Newport	Ender and Kelly (2005)
Seed Yield (kg/ha)	Yd21	2	151.2	−46.88	DOR 364 × BAT 477	Diaz et al. (2017)
	Yd71	7	35.1	−36.91	DOR 364 × BAT 477	Diaz et al. (2017)
	Yd72	7	47.8	−97.3	DOR 364 × BAT 477	Diaz et al. (2017)
	syMO14	3	113.7	−153.6	BK004-001 × H68-4	Sandhu et al. (2018)
	syMO16a	7	10.6	−170.9	BK004-001 × H68-4	Sandhu et al. (2018)
	syMO16b	8	0.5	−140.2	BK004-001 × H68-4	Sandhu et al. (2018)
	SY10v1	10	41	−178.77	SER48 × Merlot	Hoyos-Villegas et al. (2016)
	SY3v3	3	53	−155.91	SER48 × Merlot	Hoyos-Villegas et al. (2016)
	SY7v3	7	51	−197.46	SER48 × Merlot	Hoyos-Villegas et al. (2016)
	SY7v4a	7	68	−178.85	SER48 × Merlot	Hoyos-Villegas et al. (2016)
	SY7v4b	7	67	−97.54	SER48 × Merlot	Hoyos-Villegas et al. (2016)

### Model for genomic selection

The model used to determine the marker effects in genomic selection is shown in Eq. 3:

3 $$y = X\beta + Zu + \varepsilon$$

where $\mathrm{u} \sim N(0, K{{\sigma }^{2}}_{u})$, ***y*** is the phenotypic value of a trait, *X* is the design matrix for the fixed effects $\beta$, *Z* is the design matrix for random effects *u*, and $\varepsilon$ is the residual error. The R package ‘rrBLUP’ version 4.6.1 (Endelman 2019) using the function mixed.solve was used to calculate the marker effects, or fixed effects $\beta$. The calculated marker effects were then input into the.*qug* file as locus effects. The training population consisted of the parental populations that were generated via SimuPop version 1.1.8 (Peng and Kimmel 2005). Thus, the size of the training population was 15, 30, 60, and 100, corresponding to the different parental population sizes for the different simulations.

### Simulating linkage disequilibrium

Linkage disequilibrium (LD) can be defined as a non-random association between alleles found at different loci (Flint-Garcia et al. 2003). By default, QU-GENE will generate populations in Hardy–Weinberg equilibrium with little to no LD. This is an issue for simulating genomic selection since adequate LD is necessary for markers to be linked to QTLs. To generate LD in our simulated populations, the forward-in-time simulation tool SimuPOP was used. SimuPOP is implemented in python. The program can be used to evolve a population over time in silico. By allowing a population to undergo natural selection via SimuPOP, populations with substantial LD could be obtained. The population generated from SimuPOP was converted to the QU-GENE format via R. Analysis of LD in the population was also performed in R, using the LD.Measures() function in the package ‘LDcorSV’ (version 1.3.3) and an LD heatmap was generated using the function LDheatmaps() in the package ‘LDheatmap’ (version 1.0.4). The initial and final LD patterns simulated can be found in Supplementary Figure 1.

### Handling simulation output data

QuLinePlus produces several output files that can be used to estimate the genetic gain, fixation of favorable alleles, Hamming distance, genetic variance, and effective population size. The.*fit* file reports the adjusted genotypic or fitness values for the population after each cycle. This is calculated using Eq. 5:

5 $$F_{Ad} = \frac{{F - TG_{l} }}{{TG_{h} - TG_{l} }} \times 100$$

where *F* is the fitness, *TG*_*h*_ is the highest target genotypic value, and *TG*_*l*_ is the lowest target genotypic value. The adjusted genetic gain can then be calculated as the difference from one cycle to the next, as shown in Eq. 6:

6 $$\Delta G_{Ad} = F_{Ad\left( n \right)} - F_{{Ad\left( {n - 1} \right)}}$$

where *∆G*_*AD*_ is the adjusted genetic gain, *F*_*AD(n)*_ is the adjusted fitness value after *n* cycles and *F*_*AD(n−1)*_ is the adjusted fitness value after n−1 cycles. The .*fix* file reports the percentage of fixed favorable and unfavorable alleles after each cycle. This can be used to determine the allele fixation rate. The .*ham* file reports the Hamming distance of the population after each cycle. In information theory, Hamming distance is used as a measure of dissimilarity between two strings of the same length (Li et al. 2012; Wang et al. 2015a, b). When applied to breeding programs for assessing individuals, the Hamming distance refers to the number of alleles that differ from the target genotype for all loci. In QU-GENE, the ideal genotype is defined as an individual that has accumulated of all the favorable alleles for a trait. A smaller Hamming distance would indicate an individual is closer to the target or ideal genotype, thus a lower value for the Hamming distance is more desirable. The .*var* file reports the additive genetic variance after each cycle. The reported values were converted to relative percentages where cycle 0 was used as a baseline and set to 100%. This parameter was used to assess the amount of genetic diversity in the population. The R packages ‘dplyr’ version 1.0.7 and ‘ggplot2’ version 3.3.3 were used to subset the data and generate plots (Wickham et al. 2020, 2021).

Finally, a principal component analysis (PCA) was generated for each strategy to compare the following factors: genetic gain, Hamming distance, fixation of favorable alleles, genetic variance, and effective population size. The PCA plots were created using ‘ggplot2’ version 3.3.3 in R.

## Results

### Genetic variance

The selection strategies and frameworks were first compared in terms of changes to genetic variance for the three simulated traits, days to flowering (DF), white mold tolerance (WM), and seed yield (SY). Genetic variance was represented as a relative percentage, with cycle 0 defined as 100%. Differing numbers of initial parents were also compared for each trait. The analysis of variance (ANOVA) for additive genetic variance revealed that the strategy, framework, and number of parents were all statistically significant. As expected, relative genetic variance decreased over the five cycles. For days to flowering, as the number of initial parents increased, less relative genetic variance was maintained (Supplemental Figure 2). Similar trends were observed for white mold tolerance (Supplemental Figure 3) and seed yield (Supplemental Figure 4). Genomic selection led to equal or greater genetic variance being maintained when compared to conventional breeding. Meanwhile, speed breeding resulted in lower genetic variance maintained compared to both conventional breeding and genomic selection. Interestingly, the use of genomic selection for seed yield resulted in maintenance of more genetic variance under the mass selection strategy, when compared to conventional breeding. For days to flowering, bulk breeding maintained the greatest amount of genetic variance for most scenarios. With 30 initial parents under genomic selection, the modified pedigree method maintained the most genetic variance. With 60 initial parents under genomic selection, mass selection maintained the most genetic variance.

For white mold tolerance, bulk breeding led to the greatest genetic variance maintained when the parental population size was 15. For parental population sizes of 30, 60, and 100, mass selection resulted in the most genetic variance maintained.

For seed yield, mass selection resulted in the most genetic variance being maintained for most scenarios. With 15 initial parents under conventional and speed breeding, bulk breeding led to the greatest genetic variance maintained.

### Fixation of favorable alleles and Hamming distance

**Fig. 3:**
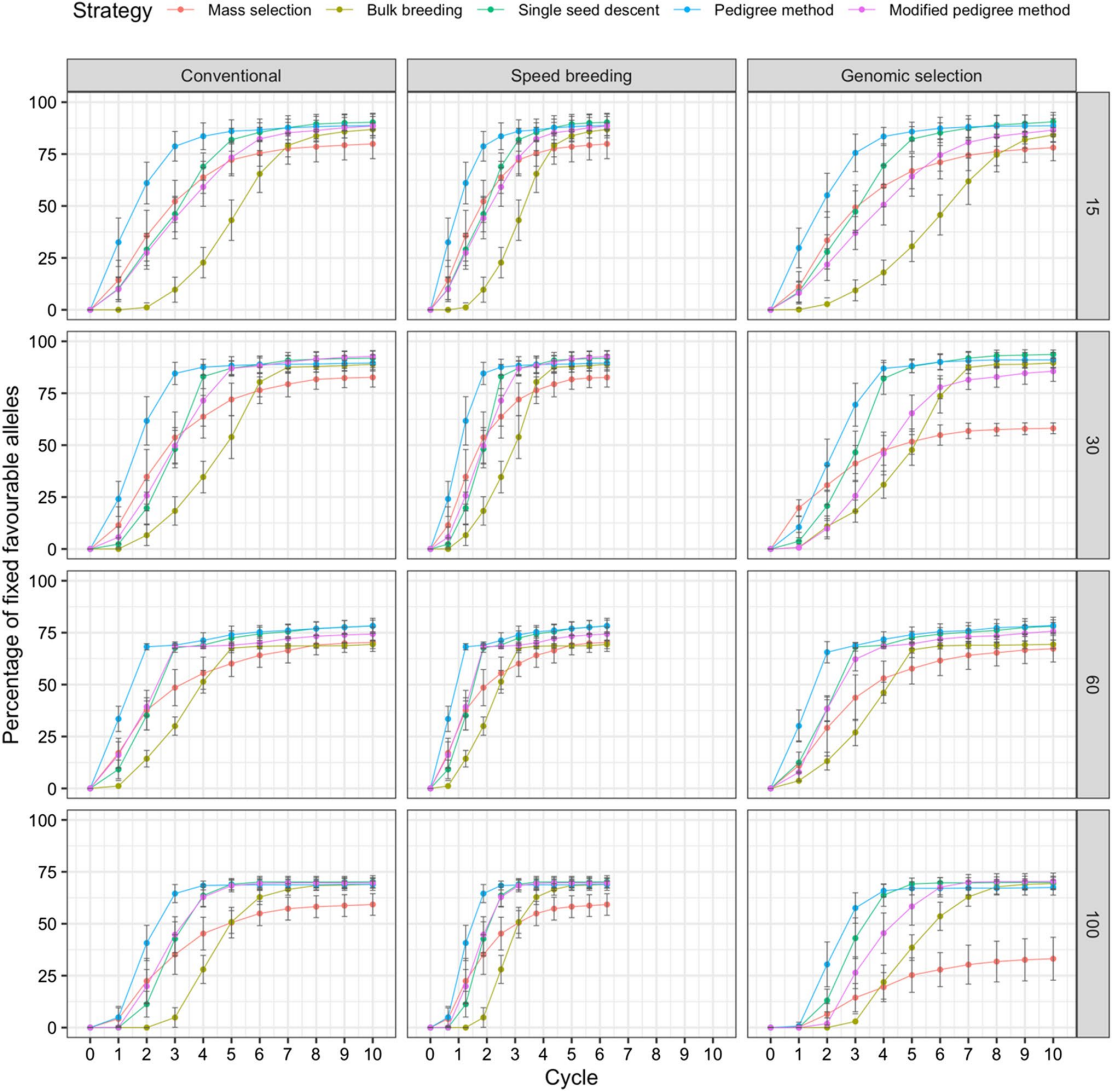
Comparison of five breeding strategies in terms of fixation of favorable alleles over 10 cycles of selection across 50 runs in a closed system. Selection for days to flowering was simulated with increasing numbers of initial parents displayed on the right and differing breeding frameworks shown at the top. Breeding strategies include mass selection, bulk breeding, single seed descent, pedigree method, modified pedigree method. Error bars indicate standard error

**Fig. 4:**
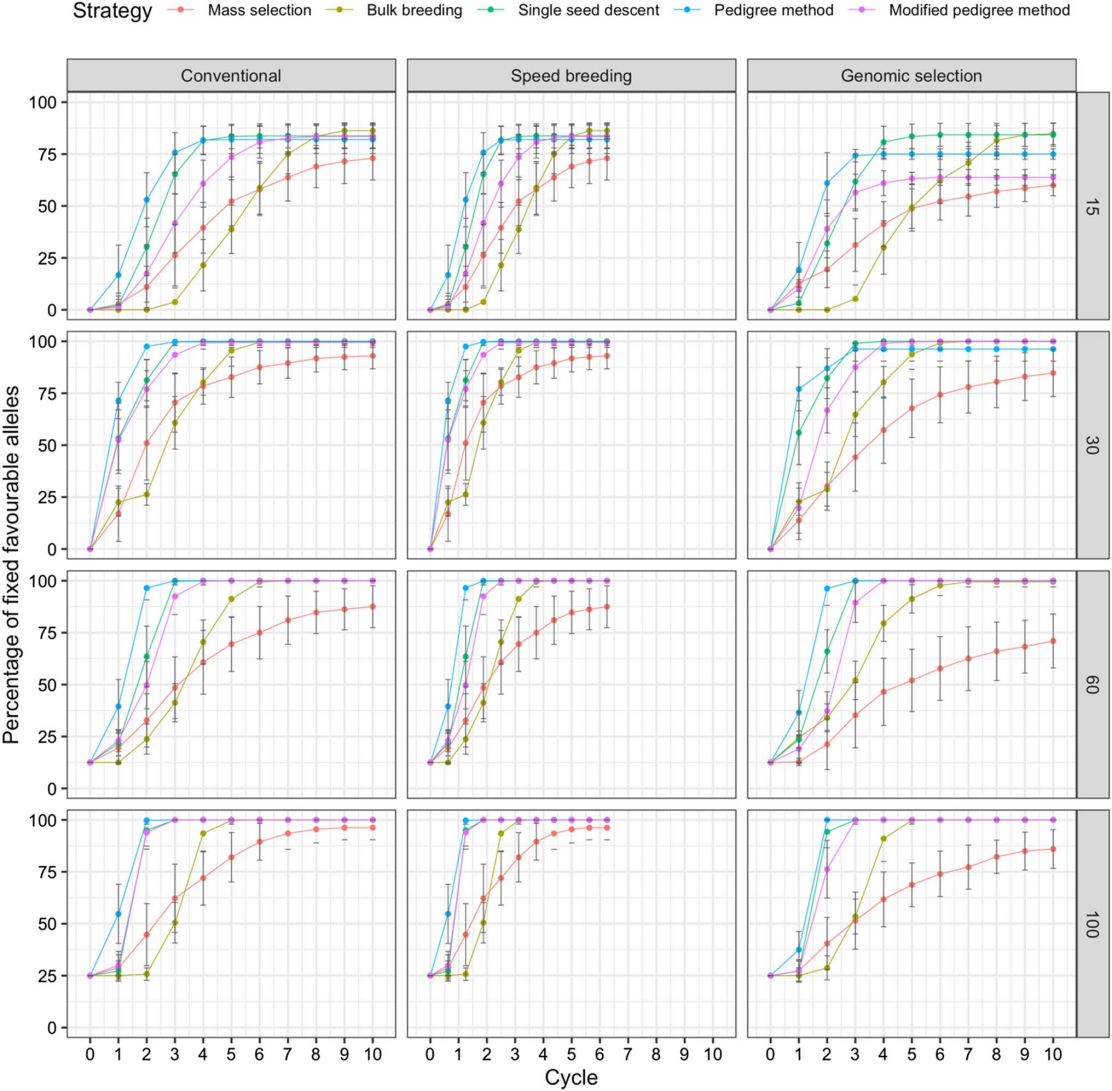
Comparison of five breeding strategies in terms of fixation of favorable alleles over 10 cycles of selection across 50 runs in a closed system. Selection for white mold tolerance was simulated with increasing numbers of initial parents displayed on the right and differing breeding frameworks shown at the top. Breeding strategies include mass selection, bulk breeding, single seed descent, pedigree method, modified pedigree method. Error bars indicate standard error

**Fig. 5:**
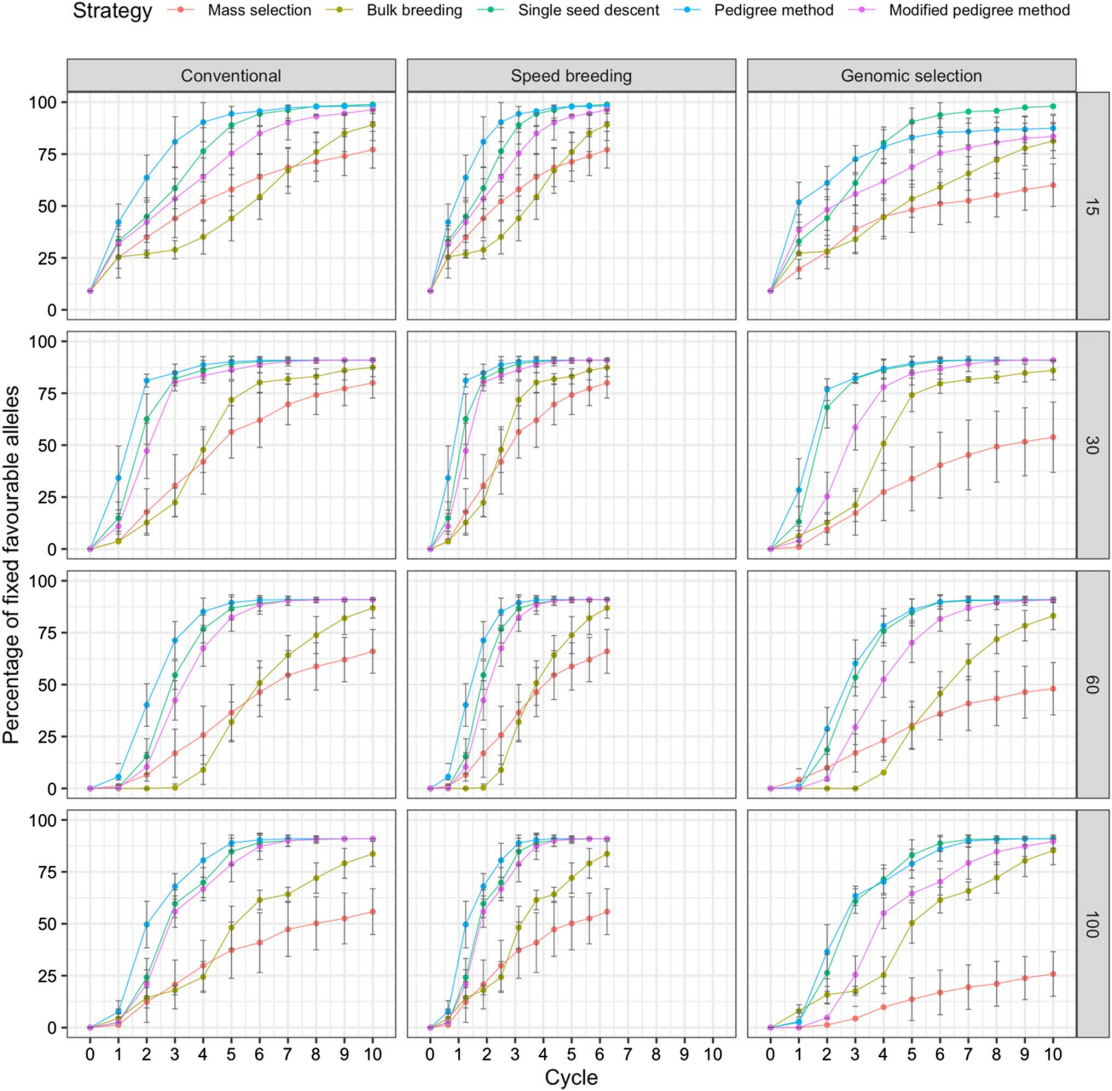
Comparison of five breeding strategies in terms of fixation of favorable alleles over 10 cycles of selection averaged across 50 runs in a closed system. Selection for seed yield was simulated with increasing numbers of initial parents displayed on the right and differing breeding frameworks shown at the top. Breeding strategies include mass selection, bulk breeding, single seed descent, pedigree method, modified pedigree method. Error bars indicate standard error

The fixation of favorable alleles was plotted over 10 cycles. Figures 3, 4 and 5 display the plots for the fixation of favorable alleles in days to flowering, white mold tolerance, and seed yield, respectively. For days to flowering (Fig. 3), as the parental population size increased, a lower percentage of alleles were fixed. Across all scenarios, the pedigree method had the fastest allele fixation rate. Mass selection had the slowest allele fixation rate and resulted in the fewest alleles being fixed. The scenario resulting in the greatest percentage of fixed alleles was single seed descent under genomic selection with 15 parents, where 93.68% of favorable alleles were fixed.

For white mold tolerance, multiple scenarios led to 100% of favorable alleles being fixed (Fig. 4). In general, as parental population size increased, a higher percentage of alleles were fixed. Under genomic selection with 100 initial parents, the pedigree method allowed for 100% of favorable alleles to be fixed in only 2 cycles. This scenario led to the greatest percentage of fixed alleles in the fewest cycles. Across all scenarios, the pedigree method had the fastest allele fixation rate.

For seed yield, a parental population size of 15 resulted in the greatest fixation of alleles (Fig. 5). The scenario resulting in the highest percentage of fixed favorable alleles was single seed descent under speed breeding with 15 initial parents, where 98.91% of alleles were fixed.

The plots for average Hamming distance are displayed in Supplemental Figures  5, 6 and 7. Overall, Hamming distance had a general decreasing trend which eventually plateaued. For days to flowering (Supplemental Figure 5), Hamming distance was higher in scenarios with larger parental population sizes, particularly for 60 and 100 parents. Across all scenarios, mass selection had the highest Hamming distance. This was especially pronounced under genomic selection when 30 and 100 parents were simulated. Conventional breeding, speed breeding, and genomic selection were all comparable, with minor differences. Under conventional and speed breeding, bulk breeding and single seed descent resulted in the lowest Hamming distance. Under genomic selection, the optimal strategy for Hamming distance depended on the parental population size. Bulk breeding, single seed descent, pedigree method, and modified pedigree method led to the smallest Hamming distance for the parental population sizes 15, 30, 60, and 100, respectively.

For white mold tolerance (Supplemental Figure 6), larger parental population sizes produced smaller Hamming distances in the selected individuals. In addition, differences between the strategies were only observed with fewer initial parents. Across all scenarios, mass selection resulted in the largest Hamming distance. The three frameworks, conventional breeding, speed breeding, and genomic selection led to similar results. With 15 initial parents, bulk breeding allowed for the smallest Hamming distance. For 30 parents under conventional and speed breeding, all strategies, except for mass selection, led to the same Hamming distance. Under genomic selection with 30 parents, bulk breeding, single seed descent, and the modified pedigree method had the smallest Hamming distance. When the parental population size was 60 and 100, the strategies, except for mass selection, resulted in the same Hamming distance after 10 cycles.

For seed yield (Supplemental Figure  7), a parental population size of 15 led to a smaller Hamming distance compared to larger parental population sizes. Similar to white mold tolerance, differences between the strategies were more noticeable with few initial parents. Mass selection consistently resulted in the largest Hamming distance across all scenarios. When comparing the Hamming distance observed in the final cycle, conventional breeding, speed breeding, and genomic selection produced similar results. It was noted that mass selection had a much larger Hamming distance under genomic selection than for the other frameworks. For 15 parents, single seed descent was the strategy that led to the smallest Hamming distance. For 30, 60, and 100 parents, the strategies, except for mass selection, resulted in the same Hamming distance.

### Genetic gain

**Fig. 6:**
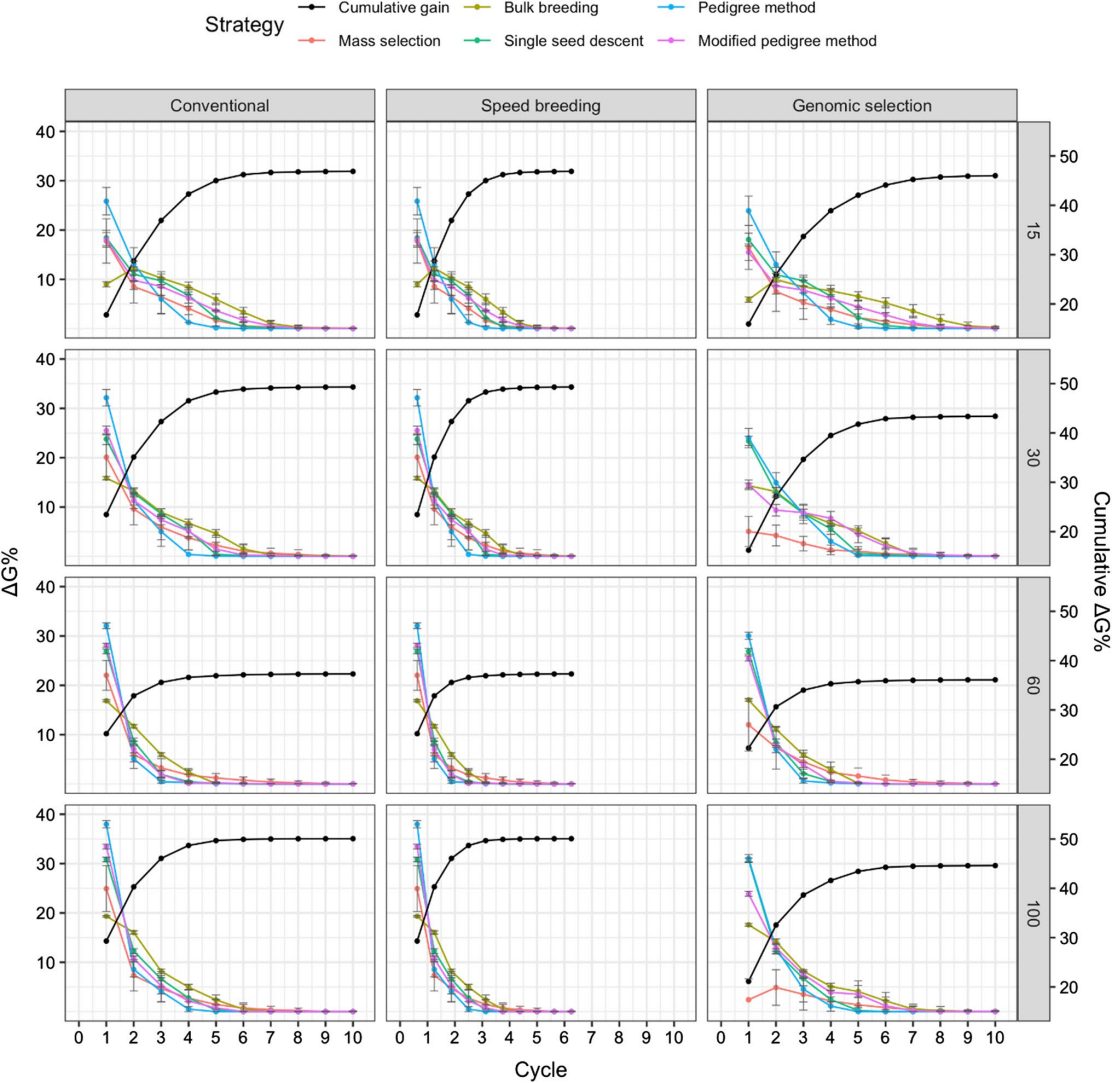
Comparison of five breeding strategies in terms of genetic gain per cycle over 10 cycles of selection averaged across 50 runs in a closed system. Selection for days to flowering was simulated with increasing numbers of initial parents displayed on the right and differing breeding frameworks shown at the top. Breeding strategies include mass selection, bulk breeding, single seed descent, pedigree method, modified pedigree method. Cumulative genetic gain averaged across strategies indicated in black on the right Y axis. Error bars indicate standard error

**Fig. 7:**
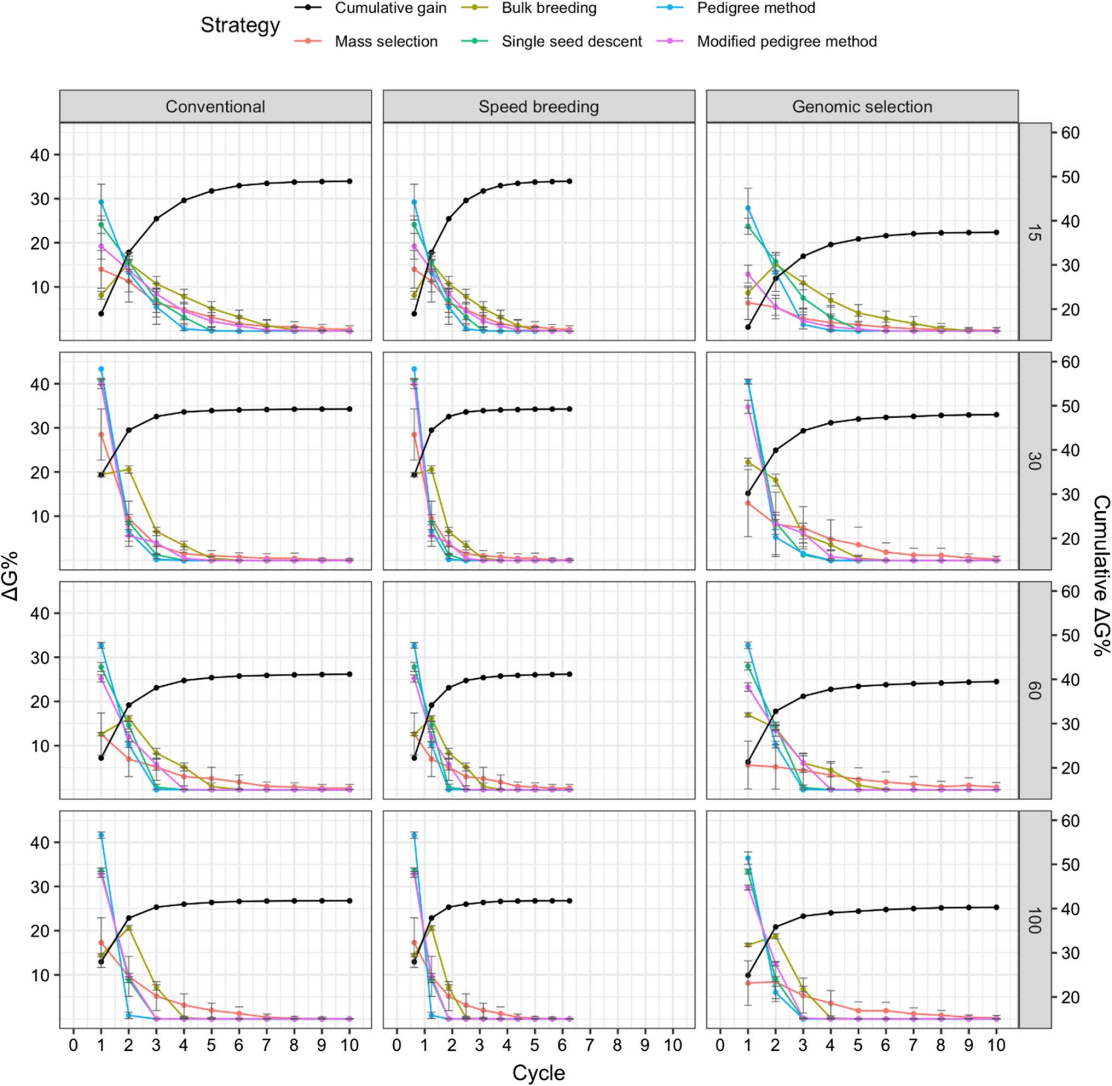
Comparison of five breeding strategies in terms of genetic gain over 10 cycles of selection averaged across 50 runs in a closed system. Selection for white mold tolerance was simulated with increasing numbers of initial parents displayed on the right and differing breeding frameworks shown at the top. Breeding strategies include mass selection, bulk breeding, single seed descent, pedigree method, modified pedigree method. Cumulative genetic gain averaged across strategies indicated in black on the right Y axis. Error bars indicate standard error

**Fig. 8:**
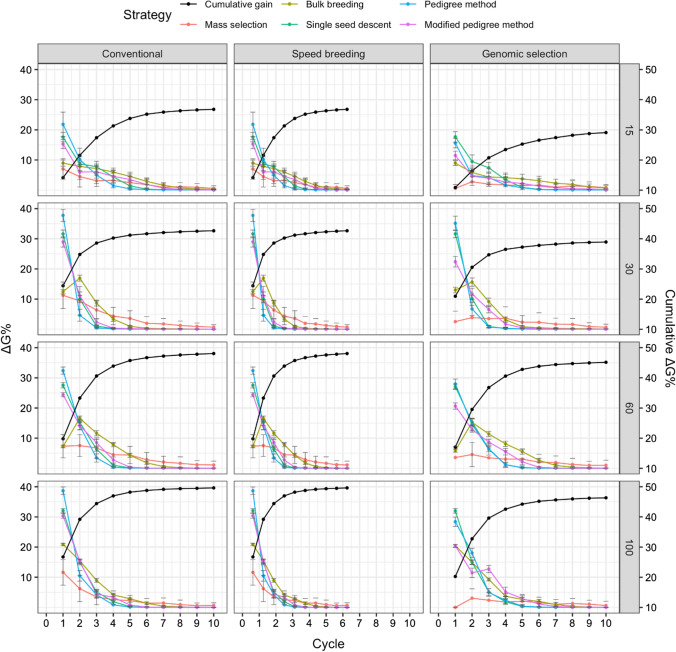
Comparison of five breeding strategies in terms of genetic gain over 10 cycles of selection averaged across 50 runs in a closed system. Selection for seed yield was simulated with increasing numbers of initial parents displayed on the right and differing breeding frameworks shown at the top. Breeding strategies include mass selection, bulk breeding, single seed descent, pedigree method, modified pedigree method. Cumulative genetic gain averaged across strategies indicated in black on the right Y axis. Error bars indicate standard error

The relative genetic gain averaged across runs was determined for each cycle for the various simulation scenarios. Figure 6 displays the trend in genetic gain for the five strategies, as well as the cumulative genetic gain averaged across strategies when days to flowering was selected. The cumulative genetic gain was greater in conventional and speed breeding compared to genomic selection for all parental population sizes. Figure 7 displays genetic gain for white mold tolerance, while Fig. 8 shows the genetic gain plot for seed yield. A parental population size of 100 led to the greatest percent cumulative genetic gain, followed by 30, 15, and 60 initial parents.

For days to flowering genetic gain (Fig. 6), the initial parental population size of 100 resulted in a maximum of 50% cumulative genetic gain, while the parental population size of 60 led to a minimum of 36% cumulative genetic gain. Conventional and speed breeding resulted in greater cumulative genetic gains compared to genomic selection.

For white mold tolerance genetic gain (Fig. 7), a parental population of 30 led to the greatest cumulative genetic gain, followed by 15, 100, and 60. Interestingly, genomic selection resulted in similar cumulative gains to conventional and speed breeding when the parental population size was 30, 60, and 100. Meanwhile, genomic selection had much lower cumulative gains than conventional and speed breeding when 15 parents were used. The parental population size of 30 resulted in a maximum of 49% cumulative genetic gain. In contrast, the parental population size 15 led to a minimum of 37% cumulative genetic gain.

For seed yield genetic gain (Fig. 8), a larger parental population size resulted in greater cumulative genetic gains, with 100 parents leading to the highest cumulative genetic gains. In general, conventional and speed breeding led to higher cumulative genetic gains compared to genomic selection. The parental population size of 100 resulted in a maximum of 50% cumulative genetic gain. Meanwhile, the parental population size of 15 led to a minimum of 29% cumulative genetic gain.

The proportion of cumulative genetic gain was determined for each cycle when averaged across all strategies (Figs. 6, 7, 8). The proportions were determined for the simulation of days to flowering. By cycle five under the conventional framework, on average the various strategies had achieved between 91 and 96% of cumulative genetic gain. Meanwhile, for speed breeding, 91–96% of cumulative genetic gain was achieved within the first three cycles. Lastly, for genomic selection, 89–98% of the cumulative genetic gain was achieved in the first 6 cycles.

**Fig. 9:**
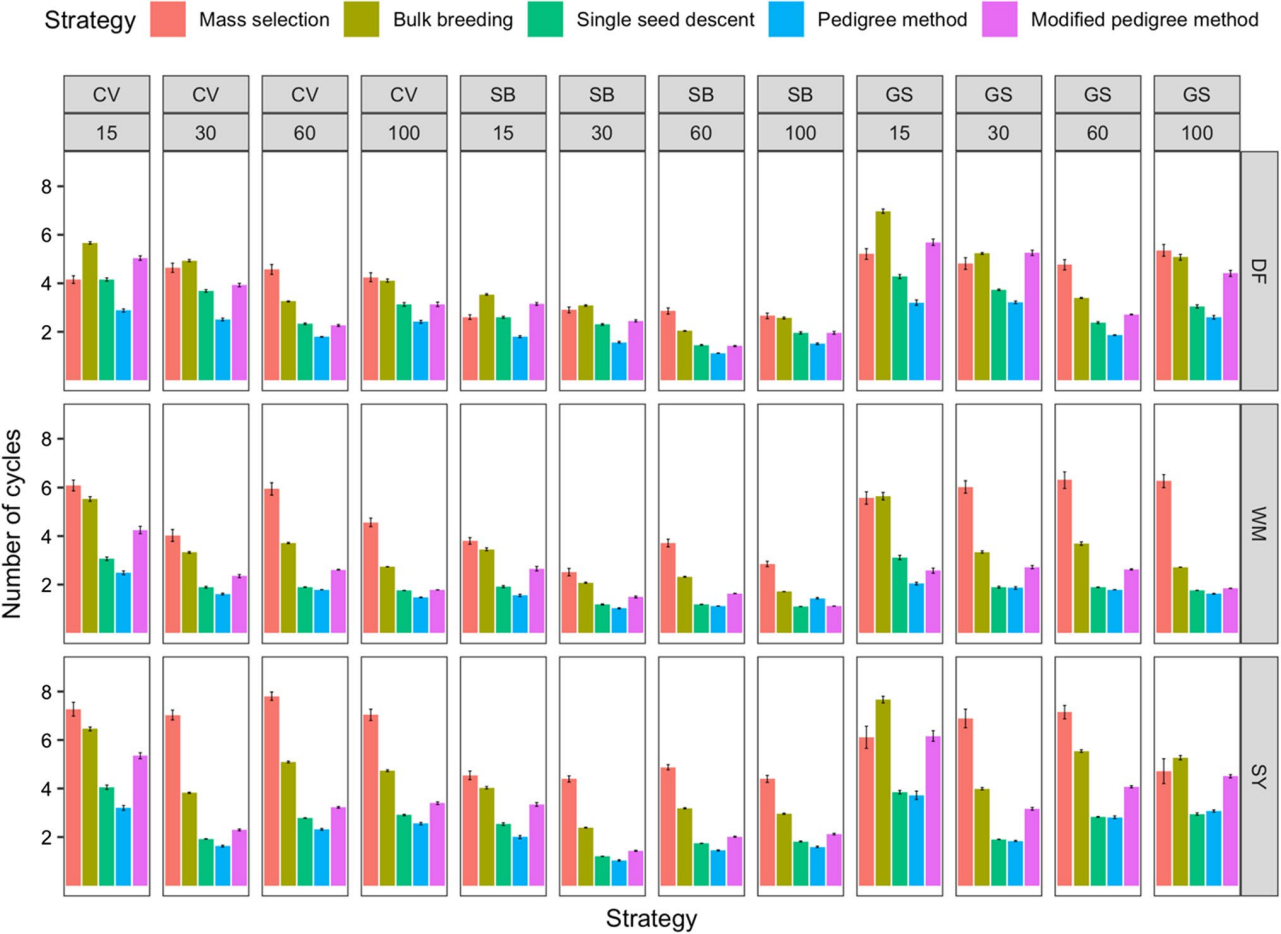
Comparison of five breeding strategies in terms of number of cycles until 95% cumulative of genetic gain for 10 cycles averaged over 50 runs in a closed system. Selected traits include days to flowering (DF), white mold tolerance (WM), and seed yield (SY). Increasing numbers of initial parents displayed on the top along with different breeding frameworks. Breeding frameworks include conventional breeding (CV), speed breeding (SB), and genomic selection (GS). Colored bars represent the breeding strategies, which include mass selection, bulk breeding, single seed descent, pedigree method, and modified pedigree method. Error bars indicate standard error

In the simulation for improving white mold tolerance, 83–97% of cumulative genetic gain was achieved by cycle 3 for under the conventional framework. Meanwhile, speed breeding led to 83–97% of cumulative genetic gains in the first 2 cycles. 93–96% cumulative gains were observed in genomic selection. Figure 9 shows the number of cycles required for 95% cumulative ΔG. On average, 3.31 cycles were necessary to achieve 95% cumulative ΔG. The scenario requiring the fewest cycles to obtain 95% cumulative ΔG was dependent on the trait. For days to flowering, the pedigree method under speed breeding with 60 parents required only 1.12 cycles to achieve 95% cumulative ΔG. For white mold tolerance, the pedigree method under speed breeding with 30 initial parents required 1.02. For seed yield, the pedigree method under speed breeding with 30 initial parents allowed for 95% cumulative ΔG to be obtained in 1.04 cycles.

**Fig. 10:**
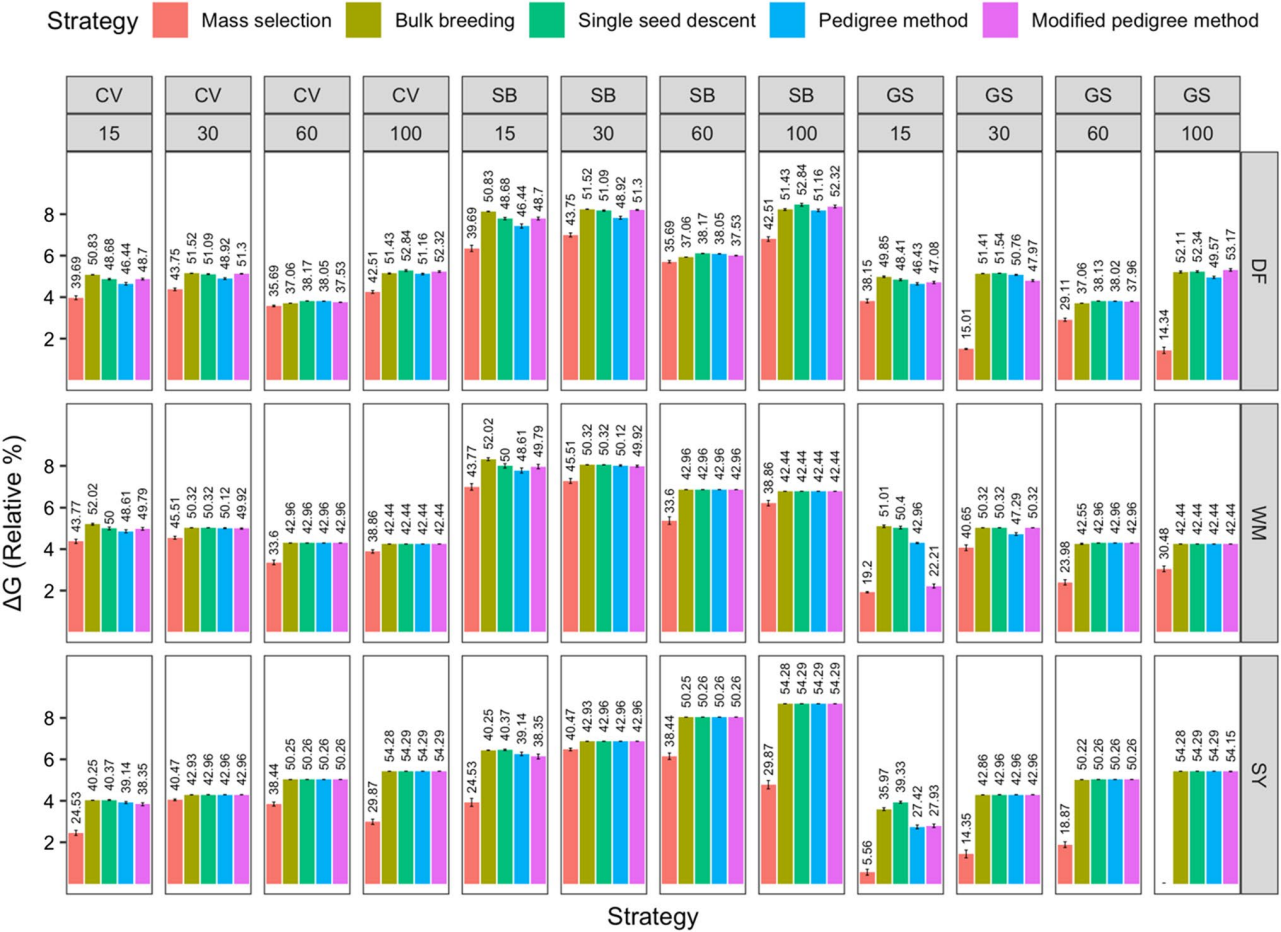
Comparison of five breeding strategies in terms of relative genetic gain per cycle across 10 cycles averaged over 50 runs in a closed system. Selected traits include days to flowering (DF), white mold tolerance (WM), and seed yield (SY). Increasing numbers of initial parents displayed on the top along with different breeding frameworks. Breeding frameworks include conventional breeding (CV), speed breeding (SB), and genomic selection (GS). Colored bars represent the breeding strategies, which include mass selection, bulk breeding, single seed descent, pedigree method, and modified pedigree method. Error bars indicate standard error. Values above bars indicate the total cumulative genetic gain (%) at the end of the simulation

The average ΔG per cycle was determined for all scenarios (Fig. 10). On average, 5.25% ΔG could be obtained per cycle. The scenario resulting in the greatest ΔG per cycle varied depending on the trait being selected. For days to flowering, single seed descent with 100 initial parents under speed breeding led to 8.45% ΔG per cycle. For white mold tolerance, bulk breeding with 15 initial parents under speed breeding resulted in 8.32% ΔG per cycle. For seed yield, single seed descent, pedigree method, and modified pedigree method with 100 initial parents under speed breeding each led to 8.69% ΔG per cycle.

### Principal component analysis (PCA)

**Fig. 11:**
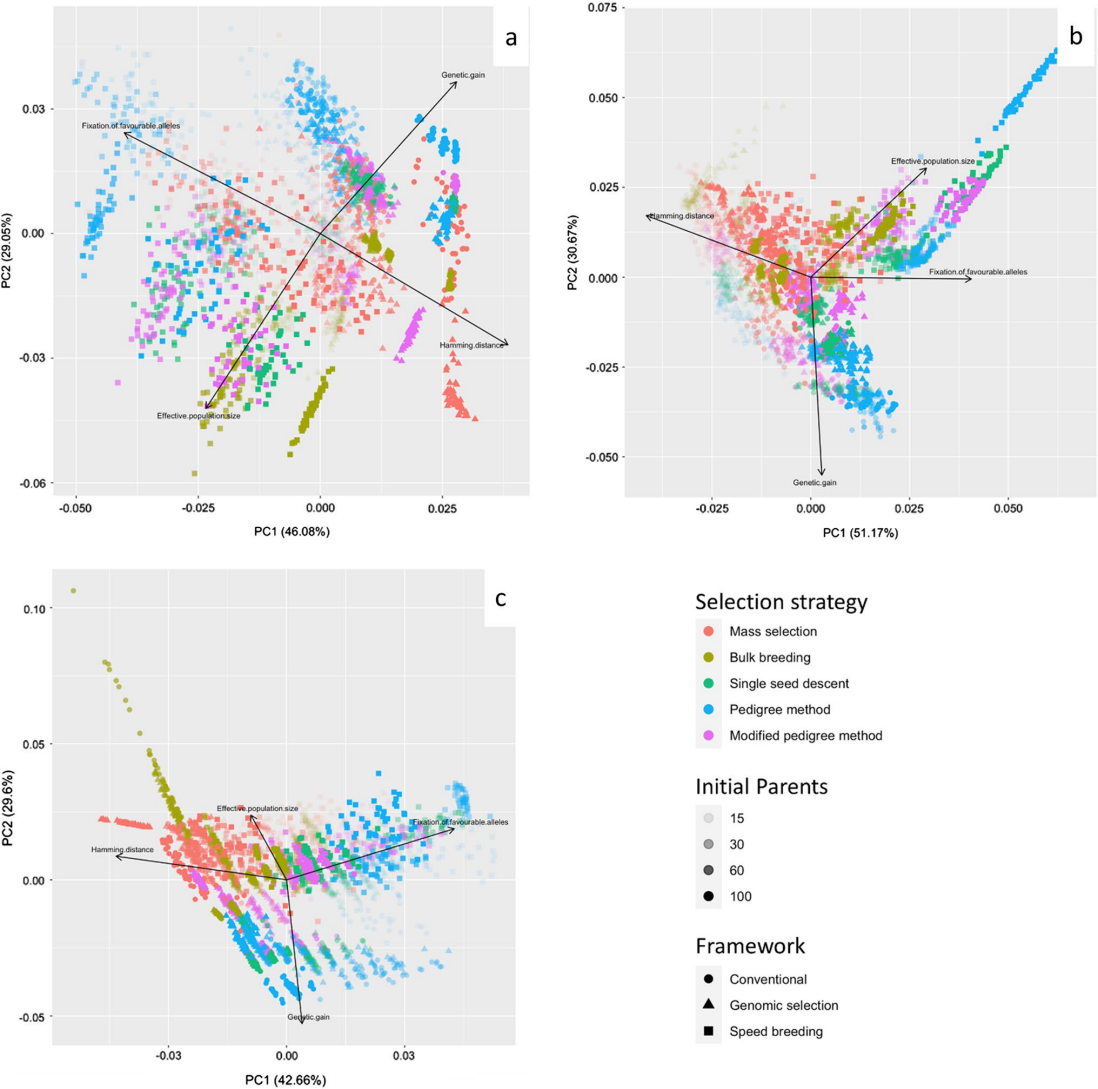
Principal component analysis (PCA) plot of genetic gain across five breeding strategies, three breeding frameworks and four initial parent population sizes. (**a**) Days to flowering, (**b**) white mold tolerance and (**c**) seed yield were selected in simulated populations with increasing parental population sizes represented by different shapes. Breeding strategies include mass selection, bulk breeding, single seed descent, pedigree method, and modified pedigree method, and are distinguished by color. Vectors specify the direction and strength of genetic gain variables. The first two principal axes explained 75.1% of the variance

Principal component analyses were conducted to examine patterns in simulation outputs immediately after the first cycle, where each run is represented by a single point on the biplot. Results are shown in Fig. 11 with eigenvector loadings of various population and quantitative genetics statistics, such as effective population size, fixation of favorable alleles, hamming distance and genetic gain. These statistics were evaluated on the simulated populations under selection for three traits (days to flowering, white mold tolerance, and seed yield) with varying levels of initial parental population sizes, heritability, breeding frameworks and selection strategies.

PCA biplots also included genomic selection and speed breeding as breeding method alternatives to conventional, and all of these contained various selection strategies (bulk, mass, pedigree, modified pedigree and single seed descent). For days to flowering (Fig. 11), a large linear-like cluster representing a parental population size of 100 can be observed to the right of the PCA plot between the eigenvectors for genetic gain and Hamming distance. At the extreme of the eigenvector for Hamming distance, was the cluster of runs for mass selection under genomic selection. There was a cluster for pedigree method with 100 parents under conventional breeding in the direction of the eigenvector of the genetic gain. At the extreme of the eigenvector for effective population size, there was a cluster corresponding to bulk breeding with a parental population size of 100 under speed breeding. A cluster representing the pedigree method with 15 and 30 parents under speed breeding formed in the extreme of the eigenvector for the fixation of favorable alleles. Between the eigenvectors for fixed favorable alleles and genetic gain, a cluster corresponding to the pedigree method under genomic selection and speed breeding was found. A cluster representing both single seed descent and the modified pedigree method was located closer to the center of the plot along the axis of the genetic gain vector. Between the eigenvectors for fixed favorable alleles and effective population size, a cluster consisting of multiple strategies including mass selection, the pedigree method, single seed descent, and the modified pedigree method was found.

For white mold tolerance, the first two principal components accounted for 81.8% of the variance (Fig. 11). Notably, there were fewer distinct clusters that formed, with most points concentrated in the center of the plot. To the extreme in the direction of the effective population size eigenvector, there was a line-like cluster representing the pedigree method under speed breeding. Between the eigenvectors for effective population size and fixed favorable alleles, there was a cluster consisting of single seed descent and the modified pedigree method under speed breeding. Between the eigenvectors for Hamming distance and effective population size, there were many points corresponding to mass selection. Points reflecting all the strategies were dispersed between the vectors for Hamming distance and genetic gain, with a larger parental population size concentrated toward the center of the plot. In the most extreme of the vector for genetic gain, there were many points representing the pedigree method with 15 and 30 parents under conventional breeding.

For seed yield (Fig. 11), the two major principal components explained 72.3% of the variance. In the outermost region of the plot, there were a number of points representing bulk breeding with 100 parents under conventional breeding between the vectors for Hamming distance and effective population size. Toward the center of the plot, there were clusters for bulk breeding that corresponded to speed breeding and genomic selection, as well as mass selection. There was a distinct cluster for mass selection with 100 parents under genomic selection that was in the direction of the Hamming distance eigenvector. In the direction of the genetic gain eigenvector, there was a cluster corresponding to the pedigree method under conventional breeding. Meanwhile, there was a sparse cluster along the fixed favorable alleles eigenvector, which consisted of the pedigree method, single seed descent, and the modified pedigree method. More points representing single seed descent and the modified pedigree method with 100 parents were found in the center of the plot. In the extreme of the fixed favorable allele eigenvector were points corresponding to the pedigree method with 30 parents under speed breeding.

## Discussion

### Comparison of selection strategies

The selection strategies showed different responses for each scenario simulated and depended on the trait being selected. For the trait days to flowering, the scenario utilizing single seed descent led to the highest genetic gain per cycle. For white mold tolerance, the breeding scenario using bulk breeding resulted in the greatest gain achieved for each cycle. For seed yield, the scenario producing to the greatest genetic gain per cycle relied upon single seed descent, the pedigree method, or the modified pedigree method. Interestingly, for all three traits, the pedigree method required fewer cycles until 95% cumulative genetic gain, meaning it may have been more efficient, but the genetic gains achieved were smaller.

Limited studies have been conducted in common beans to compare selection strategies. However, researchers have investigated the use of different selection strategies in soybean breeding. One particular study demonstrated that for the selection of yield, the highest performing lines were obtained via the pedigree method, while single seed descent produced the highest mean seed yield. The authors also found that bulk breeding was impractical for soybean breeding (Djukic et al. 2011). In contrast, a separate study conducted on soybean breeding found that bulk breeding was the most effective for obtaining the highest yielding individuals, while the pedigree method was ideal for less complex traits (Khosla 2019). The authors noted that bulk breeding was better suited to cases where breeding materials are abundant, and in cases with limited resources, pedigree may be the better choice. The results of our simulation study, which was conducted in the common bean, closely reflect previous findings in soybean breeding. Specifically, when it came to seed yield with few breeding materials, it was found that single seed descent, the pedigree method, and the modified pedigree method resulted in the greatest genetic gains. Empirically, Urrea and Singh (1994) tested the performance of mass selection, F_2_-derived family selection and single seed descent in an interracial population from the cross between ICA Pijao x Pinto UI114. In their experiment, the authors found that the mean seed yield when testing lines derived from single seed descent was the lowest and highest when derived using the pedigree method. When dealing with traits of moderate heritability (canopy architecture), the authors found a higher frequency of desirable lines using the mass selection and single seed descent. The authors found that the mean of a relatively high heritability trait (days to maturity), was most shifted using single seed descent and mass selection, resulting in more early maturity lines. These results are consistent with our simulations and confirm that, methods that delay line derivation to late generations are more advantageous when dealing with traits of low heritability. In contrast, selection methods that focus on performing early generation selection can result in greater gains when heritability is moderate or high.

### Comparison of breeding framework

Three different breeding frameworks were compared in this study. These included conventional breeding, speed breeding, and genomic selection. Conventional breeding was used as a baseline for the other two frameworks to determine how implementable they are in future breeding programs. Based on the results, speed breeding led to the greatest genetic gain achieved. It also led to the fixation of favorable alleles in the shortest time. Considering the breeder’s equation, where L, the years per cycle, was greatly reduced, this outcome was to be expected. From the simulation, it was revealed that genomic selection had a similar performance to conventional frameworks. The effectiveness of genomic selection greatly depends on the prediction accuracy, as well as the time and costs saved by replacing phenotyping with genotyping. While prediction accuracies of genomic selection were determined, this study did not factor the time and cost savings that would be associated with the use of genomic selection. Nonetheless, genomic selection performed on a level that was similar to conventional breeding. As the main advantage with genomic selection is the opportunity to circumvent phenotyping costs, breeders may find utilizing genomic selection to be worthwhile if they have the means to perform large-scale genotyping. However, depending on model accuracy erosion rate, breeding method, and trait of interest, genomic selection may underperform conventional frameworks. Breeders considering genomic selection may also need to consider the expenses associated with establishing the optimal training population, which may require more resources (Hickey et al. 2014). In terms of prediction accuracy, Taylor (2014) reported that GS is optimized when the training population is dynamic, where the progeny of the training population is combined with the training population. In addition, GS is expected to perform poorly if training takes place in one population, but GEBV are to be obtained for a reproductively isolated population. Finally, it was noted that GS becomes less effective in each advancing generation if a static training population is to be used for predicting traits that are difficult to phenotype.

### Number of initial parents and crosses

Four different parental population sizes were investigated in this study. A full diallel crossing scheme was employed for each breeding scenario. Since a closed breeding system was simulated, the lines selected at the end of the cycle would be used as the parents of the next cycle. As a result, there was a need for fewer parents and more crosses. While this scheme was mainly used to accommodate the requirements of a closed breeding system, previous researchers have theorized that having more crosses with smaller populations is more effective. According to Bernardo (2003), Witcombe and Virk (2001) and Yonezawa and Yamagata (1978), the use of more crosses with smaller populations was more effective. At the F_**2**_ generation, a breeder with limited resources has the option to create more crosses, each with smaller populations, or create fewer crosses, each with larger populations. This assumed that no prior knowledge on the crosses were available and was found to be true for any choice of parents. In practice, plant breeders will often have information, such as the pedigree and the performance of parents. The optimal choice of parents can typically be ascertained from general and specific combining abilities, and breeders can make decisions accordingly. For simulations, where parents are not thoroughly tested for general and specific combining ability, the inclusion of more parents may influence the effectiveness of the breeding program. The simulation study presented here considered four different parental population sizes. For two of the three traits analyzed, a larger parental population size resulted in higher %Δ*G* per cycle compared to smaller parental population sizes. Since a full diallel crossing scheme was implemented, there was a greater likelihood that a high performing cross was created and later selected for. For the trait white mold, the smallest parental population size led to the greatest %Δ*G* per cycle. The total cumulative genetic gain was higher with the use of 15 parents. Under 100 parents, the initial genetic gains were quite high, but gains dropped off very quickly within the first few cycles. The white mold simulation consisted of the fewest QTLs, and 100% of the favorable alleles were fixed within the first two cycles. Thus, selection for white mold was very efficient and it’s likely that there was no genetic variance remained after the first couple of cycles in the scenario involving 100 parents. Based on the breeder’s Eq. (1), the amount of additive genetic variance will influence the genetic gain. As a result, after the first two cycles of selection under 100 parents, no additional genetic gain could be achieved. Moreover, another limitation to this study was that a closed system was simulated, meaning new genetic sources were not introduced to the population. At the time of the study, the simulation software used did not have a feature to introduce new individuals into the population, and as a result a closed system was simulated. A closed system may have also contributed to a quicker reduction in genetic variance.

### Trait heritability and number of QTLs

The three traits that were simulated had different heritability levels. Days to flowering was a high heritability trait, with a narrow-sense heritability of 0.9. White mold tolerance had a moderate heritability of 0.6, while seed yield had a low heritability of 0.3. The traits also had differing numbers of QTLs, which were included based on certain criteria and available information in the literature. The aim of the study was to simulate breeding scenarios that would closely reflect breeding programs in real life. Thus, only QTLs with reported effects were included. This is unique from previous studies, in which QTL effects were randomly drawn from a normal distribution (Ali et al. 2020; Lorenz 2013; Wang et al. 2003). For all traits, the optimal framework was speed breeding. However, the optimal strategy and number of parents was dependant on the trait being selected. For white mold tolerance, the optimal number of parents was 15, while for seed yield and days to flowering, the optimal number of parents was 100. This may be due to the number of QTLs that were included in the simulation. For white mold tolerance, only 8 QTLs were considered. Selection was likely very efficient and in a closed system, little to no genetic gain could be achieved after the first few cycles. This is reflected in Fig. 10, where the cumulative genetic gain is much lower in the scenario with 100 parents. Days to flowering considered many QTLs and seed yield had a lower heritability, meaning selection was likely not as efficient and the use of 100 parents was beneficial for obtaining high performing lines. One limitation to the use of QTLs was that their R^2^ values were not included. At the time of the simulation, QU-GENE did not have the option to input QTL R^2^ values, and as a result they were not included in this study. Another limitation was that in choosing to use reported QTLs, there was a cap on the number of QTLs that could be included, which made it challenging to simulate a genetic architecture in which many small effect QTLs contributed to a trait. Since the original intent was to simulate a genetic architecture with many small effect QTLs, the GS model rrBLUP was initially selected. Furthermore, when considering the computational demands, rrBLUP has been shown to be more efficient, making it an ideal choice. Although previous studies have found that the prediction accuracy in certain Bayesian models may be influenced by the number of QTLs such that a lower QTL number would produce a higher accuracy, other studies have found similar accuracies between rrBLUP and Bayesian models (Wang et al. 2015a, b; Lorenz 2013; Liu et al. 2018; Heslot et al. 2012). The inclusion and comparison of multiple GS models would have made this study more robust, thus the lack of GS model diversity was a limitation.

PCA plots revealed that the pedigree method often formed clusters in the direction of the eigenvector for the fixation of favorable alleles. This would suggest that the pedigree method had advantages over the other strategies. However, when considering genetic gain, the pedigree method was outperformed by single seed descent and bulk breeding for the simulation of days to flowering and white mold tolerance. This may be due to the efficiency of the pedigree method, which reduced genetic variance rapidly during early cycles of breeding. Other patterns observed from the PCA plots indicated that single seed descent and modified pedigree methods had similarities, as they would often cluster together. This was the case for most breeding scenarios when considering the genetic gain per cycle. The exception, however, was under genomic selection with 15 parents for white mold tolerance and seed yield, where single seed descent and modified pedigree differed significantly in terms of genetic gain per cycle. Lastly, mass selection would often cluster in the direction of the Hamming distance eigenvector. As higher values for a Hamming distance indicates a poor performing line, scenarios clustering in the direction of the Hamming distance eigenvector are likely to be more influenced by this factor.

## Conclusions

Breeding programs are complex and may be influenced by many factors. Computer simulations provide the opportunity to investigate multiple breeding scenarios at the same time to evaluate their effectiveness. The findings from this study show that the success of a breeding program is impacted by the strategy used, the chosen framework, and the parental population size. As well, the optimal breeding scenario depends on the trait being simulated. For a low heritability trait, a large parental population size produced the greatest genetic gain per cycle. For traits involving few QTLs, use of a small parental population size is sufficient. In terms of the optimal strategy, single seed descent was most effective for days to flowering, while bulk breeding was ideal for the selection of white mold tolerance. Finally, for the improvement of seed yield, single seed descent, the pedigree method, and the modified pedigree method are all acceptable strategies to use. Some of the limitations in this study mainly involved the inclusion of variable numbers of QTLs for each trait. QU-GENE requires a genetic map rather than a physical map. As a result, QTLs identified as physical positions could not easily be converted to a genetic distance and thus were omitted. Although many reported QTLs were used in this study, seed yield remains a complex trait comprised of many small effect QTLs that are difficult to detect, suggesting that QTLs included in this study represent a subsample of the total QTLs that contribute to the trait.

## Supplementary Information

Below is the link to the electronic supplementary material.

Supplementary file1 (DOCX 3481 KB)

Supplementary file2 (DOCX 53 KB)

## Data Availability

Data and code are available at: Github: https://github.com/McGillHaricots/peas-andlove/tree/master/Simulation-files

## Abbreviations

GS: Genomic selection
GEBV: Genomic estimated breeding value
