# Supplementary material for: Simulations of rate of genetic gain in dry bean breeding programs
Source: Theor Appl Genet. 2023 Jan 20;136(1):14. doi: 10.1007/s00122-023-04244-x (PMC9859924; doi:10.1007/s00122-023-04244-x)
Supplement: Supplementary file 1 — Supplementary file1 (DOCX 3481 KB) [file 122_2023_4244_MOESM1_ESM.docx]

## **Supplementary figures**

**
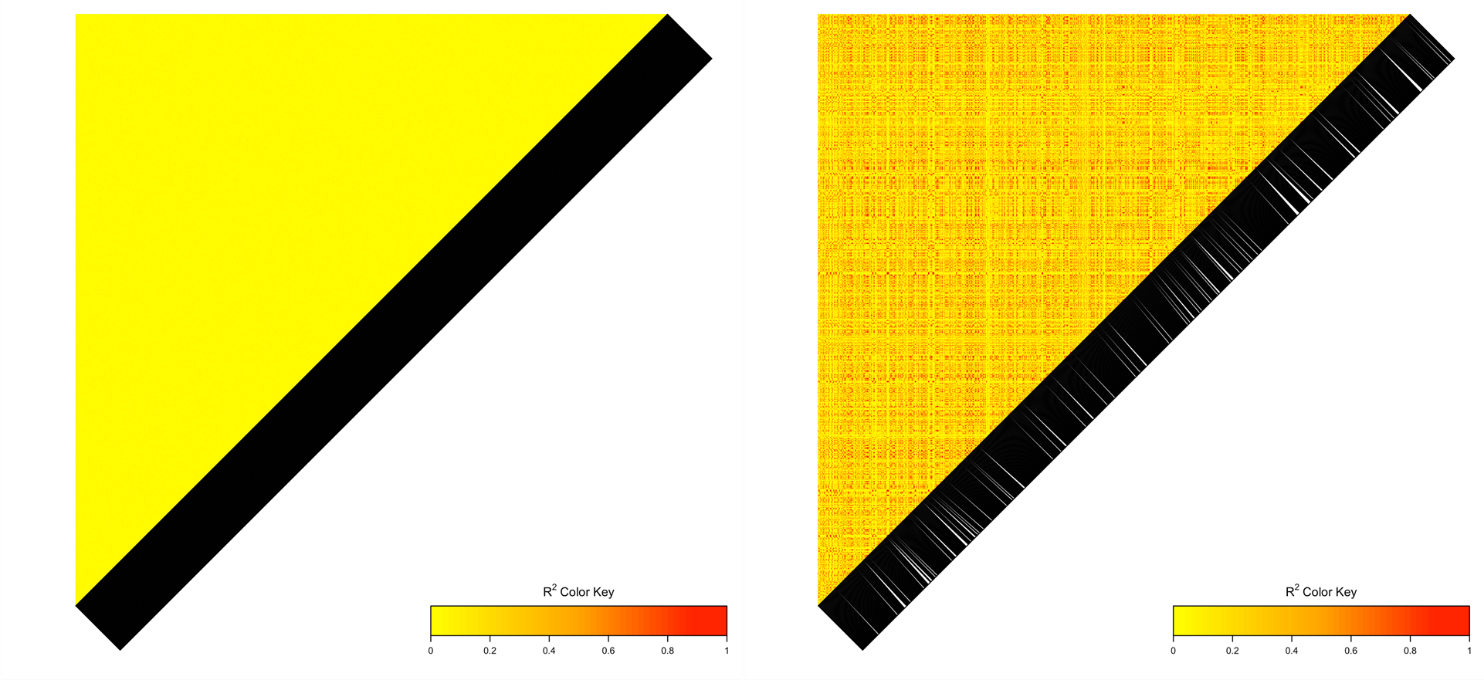
**

**Supplementary Figure 1**: LD heatmap of a QU-GENE-generated population. LD Pattern before simulation (left) and after simulation of LD (right)

**
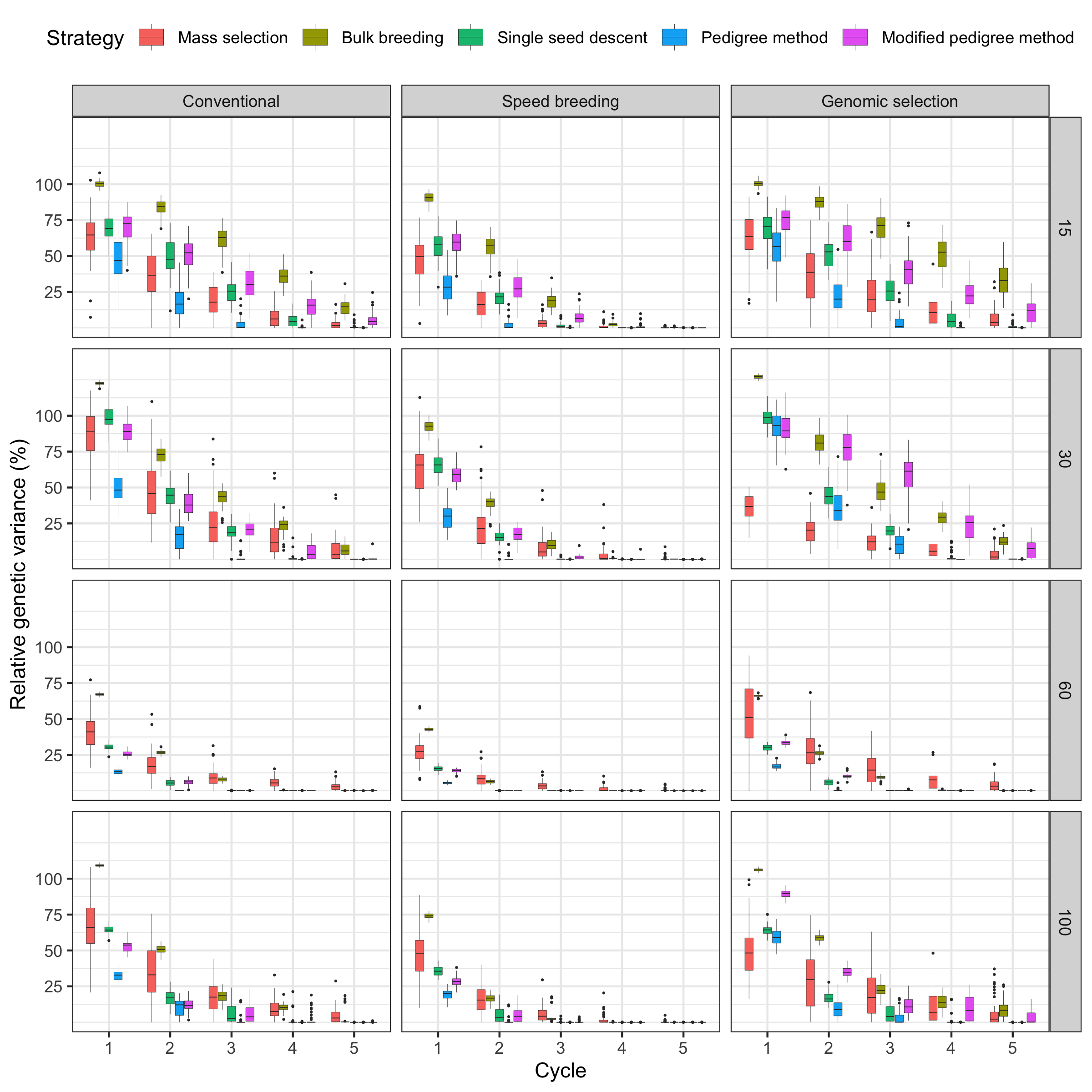
**

**Supplementary Figure 2**: Comparison of five breeding strategies in terms of genetic variance over 5 cycles of selection across 50 runs in a closed system. Selection for days to flowering was simulated with increasing numbers of initial parents displayed on the right and differing breeding frameworks shown at the top. Breeding strategies included mass selection, bulk breeding, single seed descent, pedigree method, modified pedigree method. Genetic variance is relative to cycle 0, which is 100%.

**
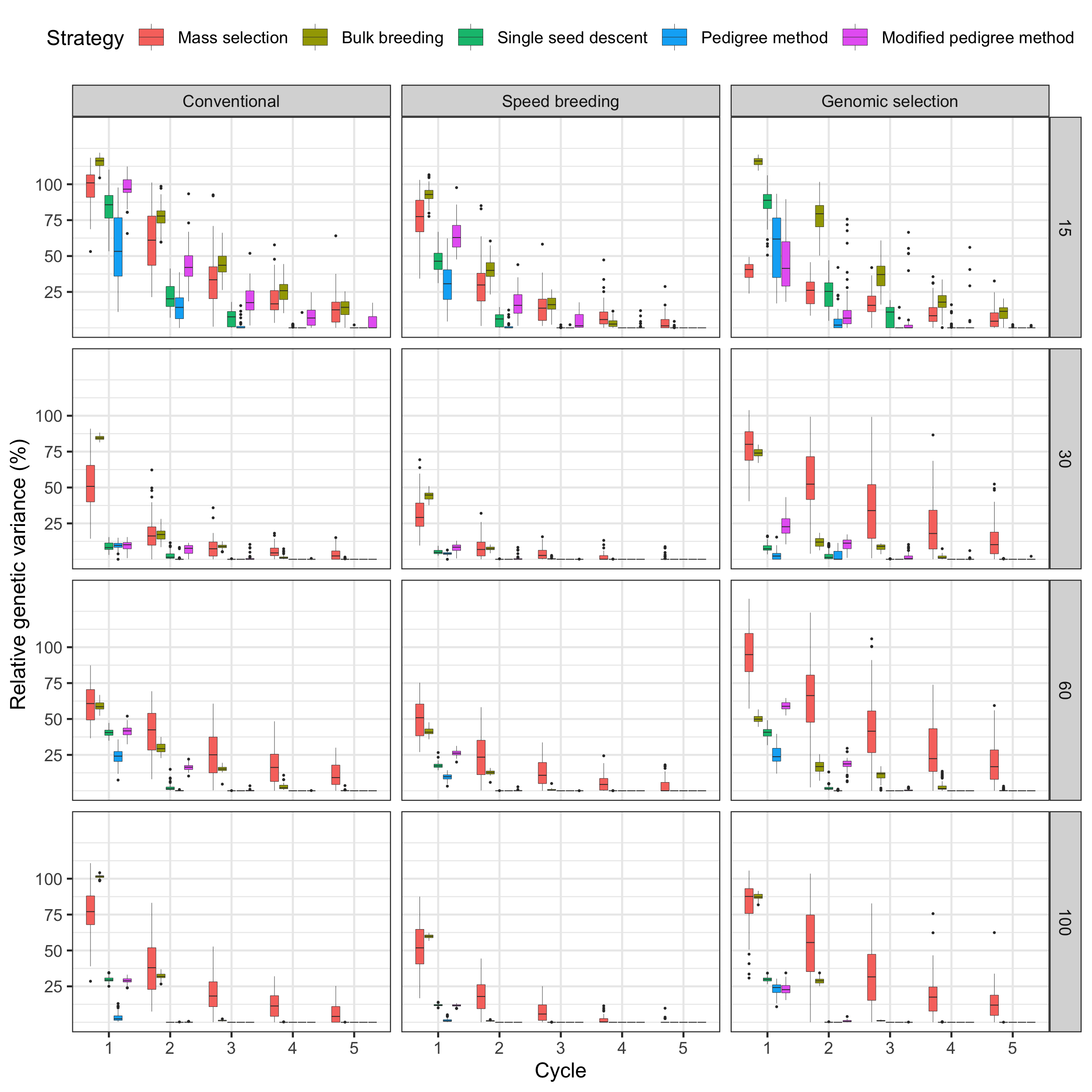
**

**Supplementary Figure 3**: Comparison of five breeding strategies in terms of genetic variance over 5 cycles of selection across 50 runs in a closed system. Selection of white mold tolerance was simulated with increasing numbers of initial parents displayed on the right and differing breeding frameworks shown at the top. Breeding strategies include mass selection, bulk breeding, single seed descent, pedigree method, modified pedigree method. Genetic variance is relative to cycle 0, which is 100%.

**
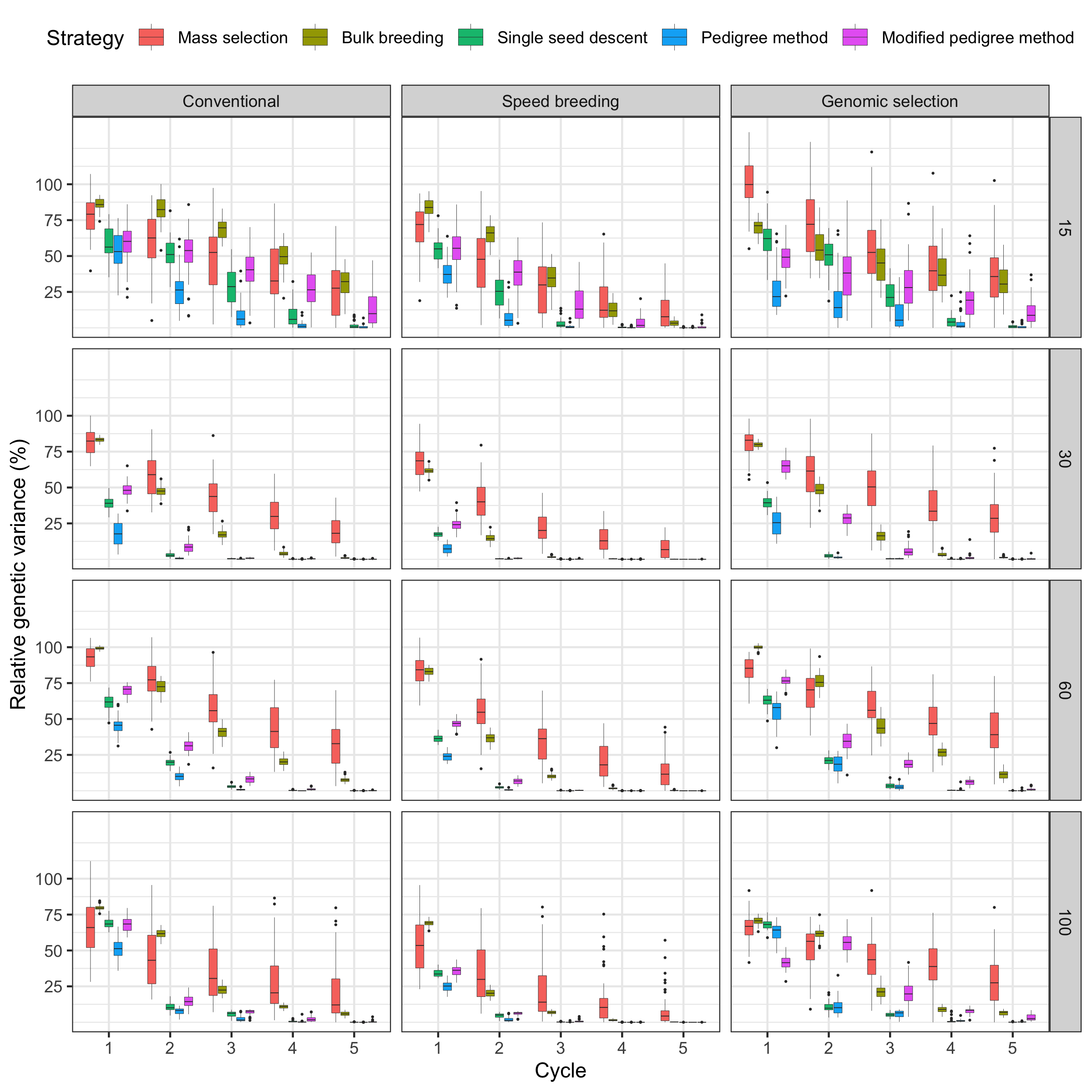
**

**Supplementary Figure 4**: Comparison of five breeding strategies in terms of genetic variance over 5 cycles of selection across 50 runs in a closed system. Seed yield selection was simulated with increasing numbers of initial parents displayed on the right and differing breeding frameworks shown at the top. Breeding strategies include mass selection, bulk breeding, single seed descent, pedigree method, modified pedigree method. Genetic variance is relative to cycle 0, which is 100%.


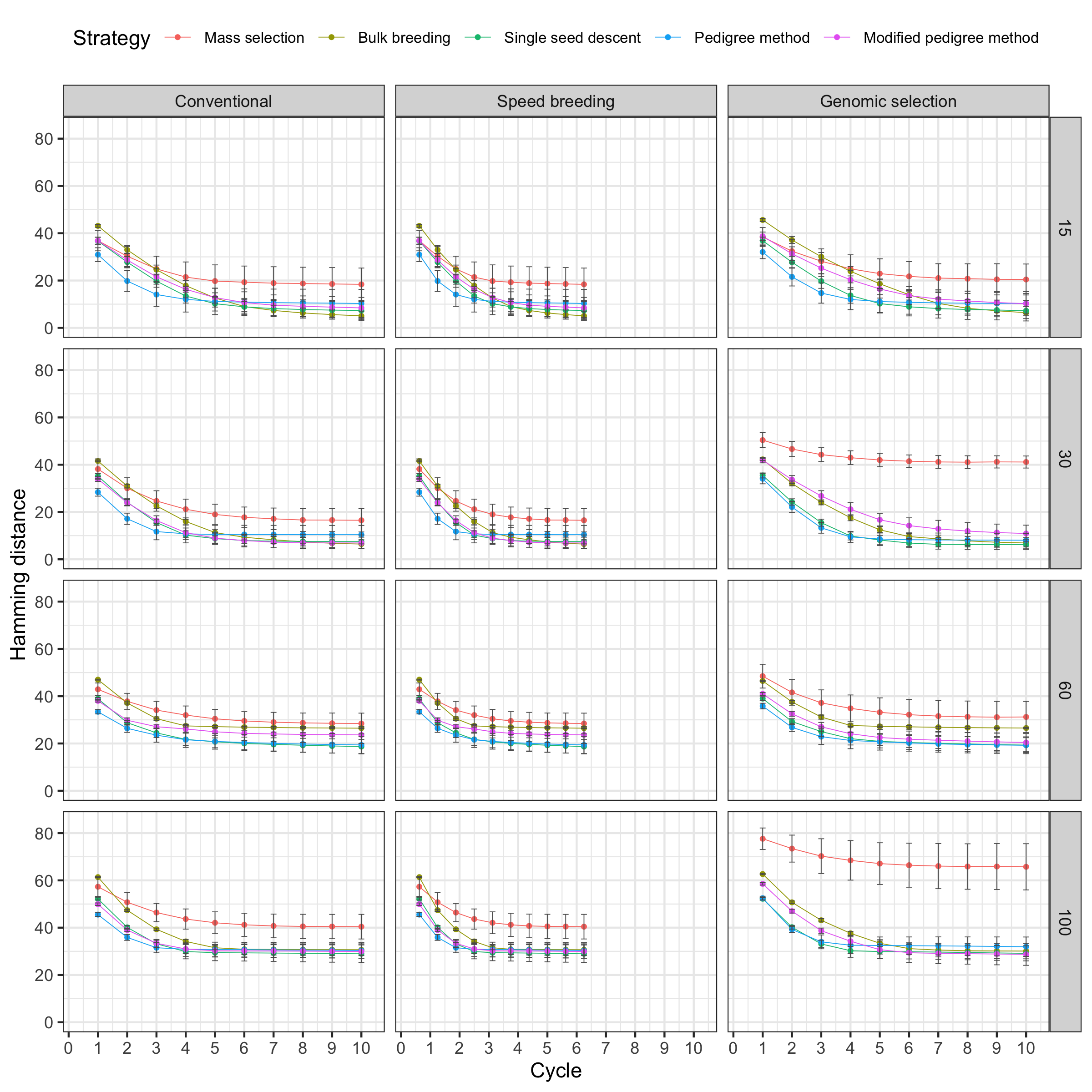


**Supplementary Figure 5**: Comparison of five breeding strategies in terms of Hamming distance over 10 cycles of selection averaged across 50 runs in a closed system. Selection for days to flowering was simulated with increasing numbers of initial parents displayed on the right and differing breeding frameworks shown at the top. Breeding strategies include mass selection, bulk breeding, single seed descent, pedigree method, modified pedigree method. Error bars indicate standard error.


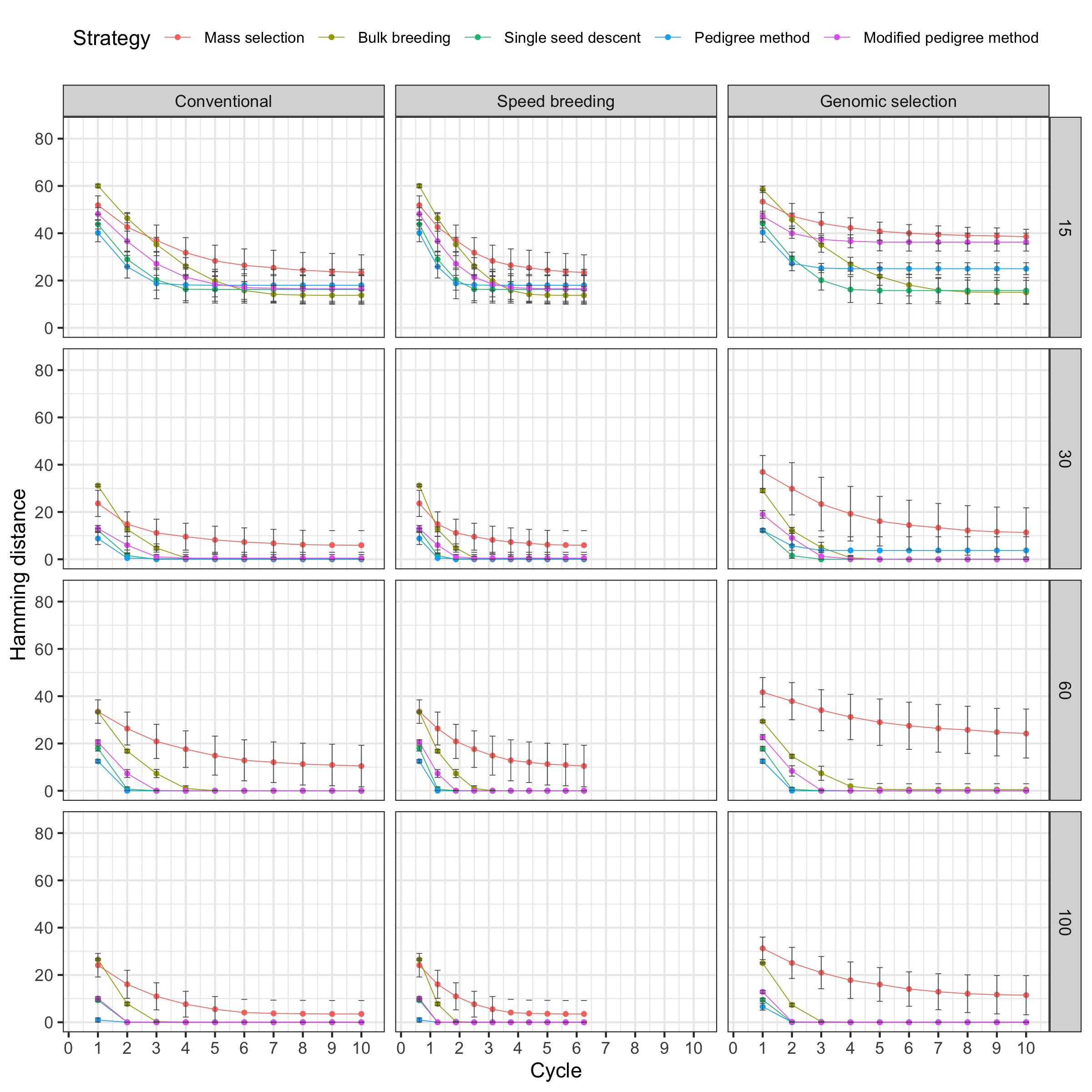


**Supplementary Figure 6.** Comparison of five breeding strategies in terms of Hamming distance over 10 cycles of selection averaged across 50 runs in a closed system. Selection for white mold tolerance was simulated with increasing numbers of initial parents displayed on the right and differing breeding frameworks shown at the top. Breeding strategies include mass selection, bulk breeding, single seed descent, pedigree method, modified pedigree method. Error bars indicate standard error.


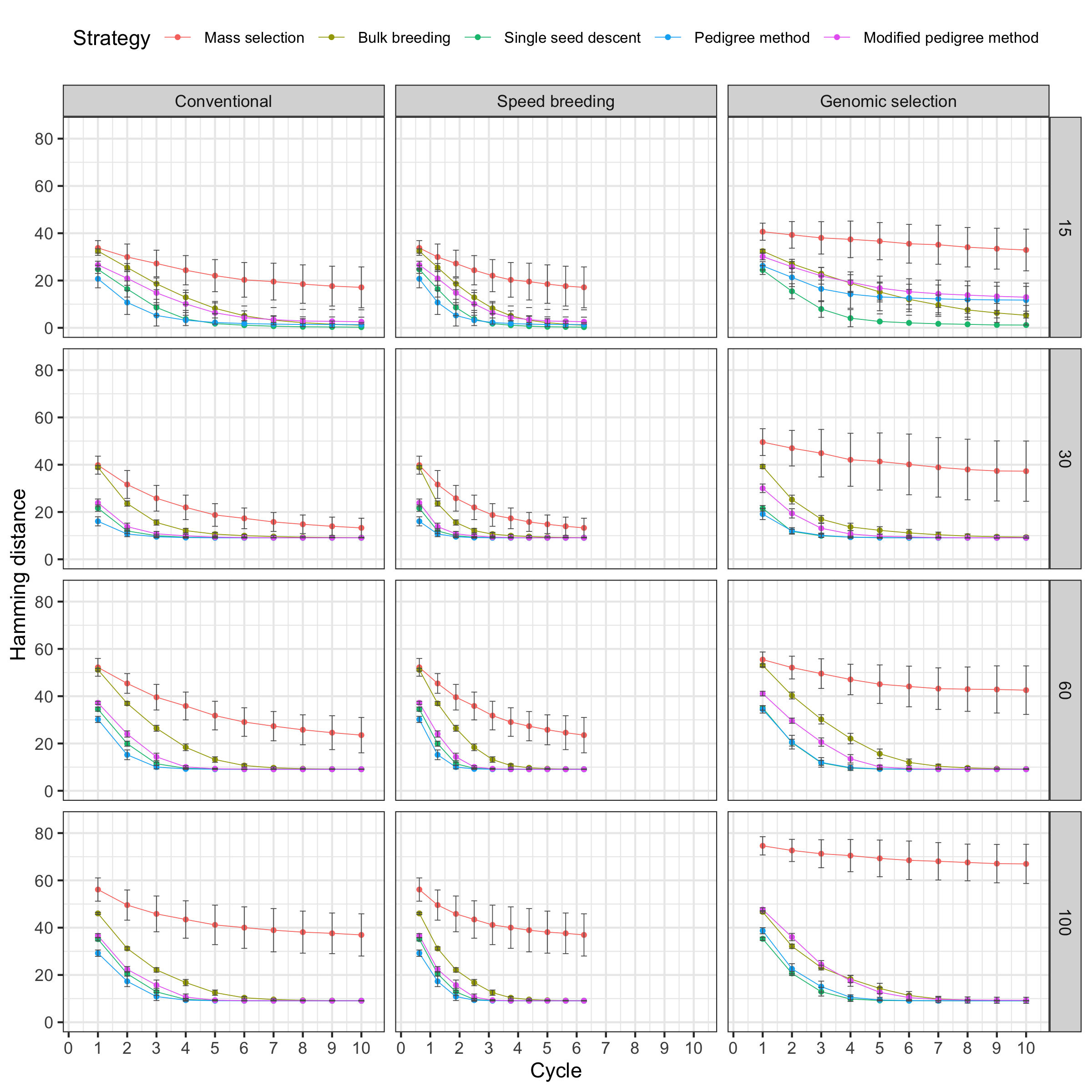


**Supplementary Figure 7.** Comparison of five breeding strategies in terms of Hamming distance over 10 cycles of selection averaged across 50 runs in a closed system. Seed yield selection was simulated with increasing numbers of initial parents displayed on the right and differing breeding frameworks shown at the top. Breeding strategies include mass selection, bulk breeding, single seed descent, pedigree method, modified pedigree method. Error bars indicate standard error.
