# Supplementary material for: Simulations of rate of genetic gain in dry bean breeding programs
Source: Theor Appl Genet. 2023 Jan 20;136(1):14. doi: 10.1007/s00122-023-04244-x (PMC9859924; doi:10.1007/s00122-023-04244-x)
Supplement: Supplementary file 2 — Supplementary file2 (DOCX 53 KB) [file 122_2023_4244_MOESM2_ESM.docx]

**Supplementary tables**

**Supplemental Table 1**: Detailed steps for the breeding strategies specified in the .qmp file
